# LncRNA *TMPO-AS1* promotes esophageal squamous cell carcinoma progression by forming biomolecular condensates with FUS and p300 to regulate *TMPO* transcription

**DOI:** 10.1038/s12276-022-00791-3

**Published:** 2022-06-27

**Authors:** Xiao-Jing Luo, Ming-Ming He, Jia Liu, Jia-Bo Zheng, Qi-Nian Wu, Yan-Xing Chen, Qi Meng, Kong-Jia Luo, Dong-Liang Chen, Rui-Hua Xu, Zhao-Lei Zeng, Ze-Xian Liu, Hui-Yan Luo

**Affiliations:** 1 Department of Medical Oncology, Sun Yat-sen University Cancer Center, State Key Laboratory of Oncology in South China, Collaborative Innovation Center for Cancer Medicine, Sun Yat-sen University, Guangzhou, 510060 People’s Republic of China; 2 Research Unit of Precision Diagnosis and Treatment for Gastrointestinal Cancer, Chinese Academy of Medical Sciences, Guangzhou, 510060 People’s Republic of China; 3 Department of Pathology, Sun Yat-sen University Cancer Center, Guangzhou, People’s Republic of China; 4 Department of Thoracic Surgery, Sun Yat-sen University Cancer Center, Guangzhou, People’s Republic of China

**Keywords:** Oesophageal cancer, Oncogenes, Long non-coding RNAs

## Abstract

Esophageal squamous cell carcinoma (ESCC) is one of the most life- and health-threatening malignant diseases worldwide, especially in China. Long noncoding RNAs (lncRNAs) have emerged as important regulators of tumorigenesis and tumor progression. However, the roles and mechanisms of lncRNAs in ESCC require further exploration. Here, in combination with a small interfering RNA (siRNA) library targeting specific lncRNAs, we performed MTS and Transwell assays to screen functional lncRNAs that were overexpressed in ESCC. *TMPO-AS1* expression was significantly upregulated in ESCC tumor samples, with higher *TMPO-AS1* expression positively correlated with shorter overall survival times. In vitro and in vivo functional experiments revealed that *TMPO-AS1* promotes the proliferation and metastasis of ESCC cells. Mechanistically, *TMPO-AS1* bound to fused in sarcoma (FUS) and recruited p300 to the *TMPO* promoter, forming biomolecular condensates in situ to activate *TMPO* transcription *in cis* by increasing the acetylation of histone H3 lysine 27 (H3K27ac). Targeting *TMPO-AS1* led to impaired ESCC tumor growth in a patient-derived xenograft (PDX) model. We found that *TMPO-AS1* is required for cell proliferation and metastasis in ESCC by promoting the expression of *TMPO*, and both *TMPO-AS1* and *TMPO* might be potential biomarkers and therapeutic targets in ESCC.

## Esophageal cancer: Regulatory RNA increases a hormone that promotes cancer

The role of a regulatory RNA in promoting esophageal squamous cell carcinoma (ESCC) has been clarified, revealing molecular details that might help in cancer diagnosis and treatment. Xiao-Jing Luo and colleagues at Sun Yat-sen University in China found that overproduction of an RNA molecule called thymopoietin-antisense RNA 1 (TMPO-AS1) in ESCC tissue samples from cancer patients was associated with shorter survival times. Overproduction of this RNA promoted proliferation and spread (metastasis) of the cancer cells. Research on details of the molecular mechanisms involved showed that the RNA ultimately activated the gene that codes for the protein hormone thymopoietin, which has previously been linked with various cancers. The authors suggest that TMPO-AS1 and thymopoietin could serve as diagnostic biomarkers of cancer and become targets for anti-cancer drugs.

## Introduction

Esophageal carcinoma (ESCA) is the 6^th^ leading cause of cancer-related mortality worldwide^1^. In China, the predominant histological subtype of ESCA is esophageal squamous cell carcinoma (ESCC), which ranks 4^th^ in cancer-related mortality^2^. Although the clinical community has achieved some diagnostic and therapeutic advances, patients with advanced ESCC have a poor prognosis due to recurrence and metastasis, leading to a 5-year survival rate of less than 20%^2,3^. Genetic abnormalities and molecular alterations play essential roles in the progression of ESCC and are potential therapeutic targets^4^. Therefore, a more comprehensive understanding of the molecular mechanism underlying ESCC progression is vital for the development of novel biomarkers and effective therapeutic targets for this disease.

Long noncoding RNAs (lncRNAs) are a class of transcripts with a length of more than 200 nucleotides and virtually no protein-coding potential^5^. LncRNAs play extensive roles in various physiological and pathological processes, including tumor initiation and progression. Recent reports have revealed diverse functional mechanisms for lncRNAs, such as acting as microRNA sponges, endogenous small interfering RNA (siRNA) precursors, or molecular scaffolds to interact with proteins or other RNAs, and even encoding short peptides^6–8^. Roles of lncRNAs in ESCC have been reported. For example, the lncRNA *DNM3OS* confers radioresistance by regulating the DNA damage response^9^, and *AGPG* regulates PFKFB3-mediated tumor glycolytic reprogramming^10^. These studies indicate that targeting lncRNAs could be a novel approach for ESCC therapy. However, further investigations into more specific roles of lncRNAs in ESCC tumorigenesis and progression are still needed.

Natural antisense (NAT) lncRNAs are classified by their genomic location with respect to the cognate protein-coding genes. The sequences of NAT lncRNAs are often partially complementary to the transcripts of their neighboring genes^6^, and NAT lncRNAs and their neighboring genes often exhibit concordant or discordant expression patterns^11^. Recent studies have shown that NAT lncRNAs function as epigenetic regulators of the expression of their cognate genes^12,13^.

In this study, we found that the upregulated NAT lncRNA *TMPO-AS1* functions as an oncogenic regulator in ESCC. *TMPO-AS1* promoted ESCC cell proliferation, G1/S progression and metastasis. Mechanistically, *TAS1* recruited FUS and p300 to the *TMPO* promoter and formed condensates in situ, which upregulated TMPO expression by increasing the deposition of H3K27ac in the promoter and activating *TMPO* transcription *in cis*, subsequently regulating the expression of CyclinD1 and metastasis-associated protein 1 (MTA1) to promote ESCC progression. Overall, this study showed the biological roles and underlying mechanisms of the *TMPO-AS1*/TMPO axis in ESCC and suggested *TMPO-AS1* as a promising prognostic indicator and therapeutic target in ESCC.

## Materials and methods

### Cell lines and cell culture

Het-1A and NE-1 cells were obtained from the American Type Culture Collection (ATCC; Rockville, MD, USA). HEK293T, KYSE30, KYSE150, KYSE180, KYSE410, KYSE510 and KYSE520 cells were obtained from the German Cell Culture Collection (DSMZ, Braunschweig, Germany). TE-1, TE-9, TE-11 and TE-15 cells were obtained from the Cell Bank of Shanghai Institute of Cell Biology (Chinese Academy of Medical Sciences, Shanghai, China). Cells were grown in basic Dulbecco’s modified Eagle’s medium (DMEM) or RPMI-1640 medium (Thermo Fisher Scientific, Waltham, MA, USA) supplemented with 10% fetal bovine serum (FBS; Invitrogen, Carlsbad, CA, USA) and 1% penicillin/streptomycin (HyClone, Logan, UT, USA) at 37 °C in 5% CO_2_. All cells were further verified via STR-PCR DNA profiling by Guangzhou Cellcook Biotech Co., Ltd. (Guangzhou, China) and tested negative for mycoplasma contamination before use.

### Human tissue specimens

Clinical samples were collected from Sun Yat-sen University Cancer Center (SYSUCC; Guangzhou, China). All patients were histologically diagnosed with ESCC. Written informed consent was obtained from all patients. The study was approved by the Medical Ethics Committee of Sun Yat-sen University.

### Cell line-derived xenograft (CDX) and patient-derived xenograft (PDX) models

To establish CDX models, ESCC cells expressing control shRNA (shCtrl) or *TAS1*-targeting sh#1 or sh#2 were injected subcutaneously into the dorsal flanks of 4-week-old female BALB/c nu/nu mice (five mice per group). Tumor growth was monitored every 3 days after transplantation using calipers. Mice bearing xenografts were euthanized at the endpoint, and tumors were weighed. PDX models were established as described previously^14^ and were used to assess the in vivo therapeutic effects of *TAS1* using ASOs. When the volume of the PDXs was ~500 mm3, we began intratumoral injections of 5 nmol of scrambled or in vivo-optimized *TMPO-AS1* ASOs (RiboBio; Guangzhou, China) per injection every 3 days, for a total of 4 consecutive doses. The target sequence is provided in Supplementary Table 1. More details are described in the supplementary methods.

### In vivo metastasis models

To establish the lung metastasis model, ESCC cells expressing luciferase and transfected with shCtrl or *TAS1*-targeting sh#1 or sh#2 were intravenously injected into 4-week-old female BALB/c nu/nu mice (six mice per group) through the tail vein. In vivo bioluminescence imaging was performed every four weeks after inoculation. The mice were euthanized 8 weeks after injection. The number of lung nodules was determined in hematoxylin-eosin (H&E)-stained serial lung tissue sections using a microscope.

To establish the popliteal sentinel lymph node metastasis model^15^, ESCC cells transfected with shCtrl or *TAS1*-targeting sh#1 or sh#2 were injected into the left footpads of 4-week-old female BALB/c nu/nu mice (six mice per group). Eight weeks after injection, the mice were euthanized, and the lymph nodes were collected. The number of metastasis-positive lymph nodes was determined. More details are described in the supplementary methods.

### Nuclear run-on (NRO) assay

The NRO assay was performed as previously described^16^. Nuclei of 4 × 10^6^ ESCC cells were freshly isolated with NP-40 lysis buffer and kept on ice before use. Nascent RNA transcripts were immunoprecipitated with an anti-BrdU antibody (Abcam, ab6326) and subjected to qPCR analysis to detect the expression of *TMPO* nascent mRNA. More details are described in the supplementary methods.

### RNA pulldown assay

*TAS1* RNA was transcribed in vitro using a MEGAscript T7 Transcription Kit (Invitrogen, USA) and labeled with a Pierce RNA 3’ End Desthiobiotinylation Kit (Thermo Scientific, USA) according to the manufacturers’ instructions. Cell lysates were prepared with Pierce IP lysis buffer (Thermo Scientific, USA). RNA pulldown was performed with a Pierce Magnetic RNA–Protein PullDown Kit (Thermo Scientific, USA) according to the instructions. Briefly, biotinylated RNA was captured on streptavidin magnetic beads and was then incubated with cell lysates at 4 °C for 6 h before washing and elution of RNA–protein complexes. The eluted proteins were subjected to WB analysis.

### RIP assay

The RIP assay was performed using a Magna RIP RNA-Binding Protein Immunoprecipitation Kit (Millipore, Bedford, MA) according to the manufacturer’s instructions. IgG isotype control and human anti-FUS antibodies (5 μg/sample, Abcam, ab70381) were used in this assay. After proteinase K digestion, the immunoprecipitated RNAs were extracted, purified, and subjected to qPCR analysis. RNA levels were normalized to those in the 10% input sample.

### Chromatin immunoprecipitation (ChIP) assay

The ChIP assay was performed using a ChIP kit from Merck Millipore (Billerica, MA, USA) according to the manufacturer’s instructions. qPCR analysis was performed to detect the DNA fragments that coimmunoprecipitated with H3K27ac. The primers specific for the *TMPO* promoter region are listed in Supplementary Table 2.

### Chromatin isolation by RNA purification (ChIRP) assay

The ChIRP assay was performed using a Magna ChIRP RNA Interactome Kit (Millipore, USA) following the manufacturer’s instructions^17^. The purified bound DNA was isolated for qRT–PCR, and proteins were analyzed by Western blotting. Probe information is included in Supplementary Table 3.

### Statistical analysis

All data are presented as the mean ± S.D. values. Student’s *t* test or one-way ANOVA and the chi-square test were performed with GraphPad Prism 8.0.1 software (GraphPad, La Jolla, CA, USA) to compare differences between groups. Correlations between the expression levels of *TMPO-AS1* and *TMPO* were analyzed using Pearson correlation analysis. Survival analysis was performed using the Kaplan–Meier method, and differences were assessed with SPSS software using the log-rank test. The levels of significance are denoted as follows: * indicates *P* < 0.05, ** indicates *P* < 0.01, *** indicates *P* < 0.001 and ns indicates not significant.

## Results

### Identification of *TMPO-AS1* as an oncogenic natural antisense lncRNA

**Fig. 1:**
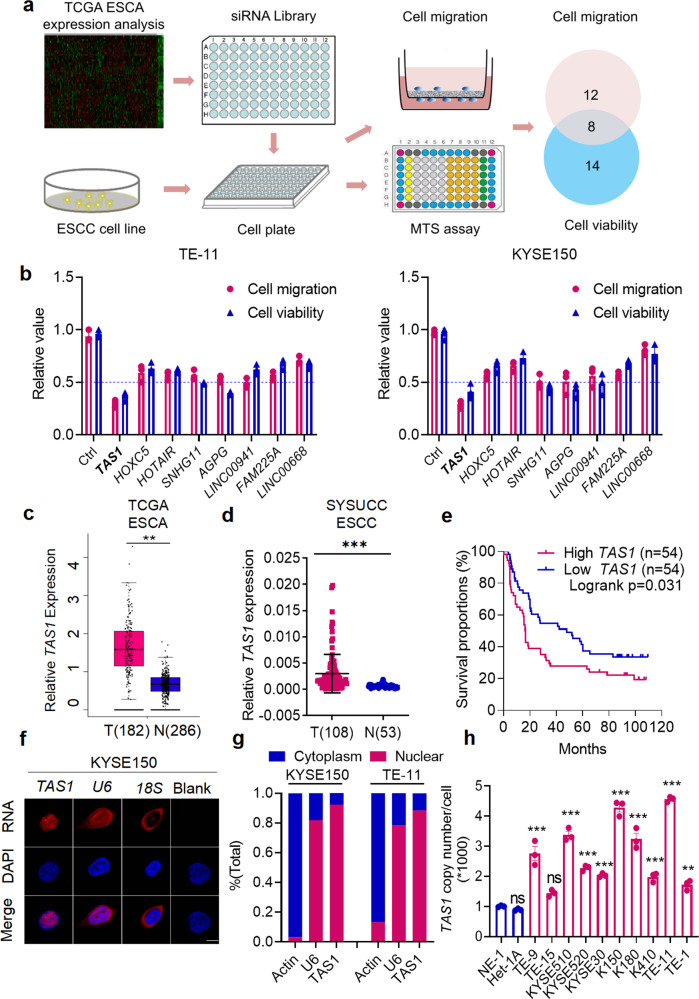
The lncRNA TMPO-AS1 (TAS1) is upregulated in ESCC and indicates poor prognosis. **a** Schematic showing the design of the screen for lncRNAs potentially involved in both cell viability and migration in ESCA. **b** Eight lncRNAs regulated both cell proliferation and migration in KYSE150 and TE-11 cells, including *TAS1*; *n* = 3 biologically independent samples. The *p* values for each group are shown in Supplementary Table 4. **c** *TAS1* expression in ESCA tissues from TCGA data. **d**, **e** *TAS1* expression and OS analysis in ESCC samples from the SYSUCC cohort. (*n* = 108, survival analysis: log-rank test, two-sided). **f** Detection of *TAS1* subcellular localization in KYSE150 cells by FISH. Scale bar: 5 μm. **g** *TAS1* expression in the nuclear and cytoplasmic fractions of KYSE150 cells and TE-11 cells, as detected using qPCR. **h** Determination of the *TAS1* copy number in ESCC cell lines and normal esophageal epithelial cell lines; *n* = 3, compared with NE1. The data are presented as the mean ± S.D. values. *P < 0.05; **P < 0.01; ***P < 0.001; ns, not significant.

We previously designed a highly efficient and specific siRNA library targeting the 50 most highly expressed lncRNAs in ESCC tumor samples compared to paired normal adjacent tissues from The Cancer Genome Atlas (TCGA) database. Using this library, we previously identified the lncRNA *AGPG*, which affects cell proliferation and glycolysis^10^. We transfected the siRNA library into two human ESCC cell lines, KYSE150 and TE-11, and performed MTS cell viability assays and Transwell migration assays to identify the lncRNAs that play essential roles in ESCC tumorigenesis and progression (Fig. 1a). Fourteen lncRNAs were found to exert promotive effects on cell proliferation, and 12 were potentially involved in cell migration; 8 of the lncRNAs were shared between both groups and might thus be involved in both cell proliferation and migration (Fig. 1a). Among these 8 lncRNAs, silencing of *TMPO-AS1* most potently attenuated ESCC cell proliferation and migration (Fig. 1b; the *p* values are shown in Supplementary Table 4). *TMPO-AS1* is an antisense lncRNA located on chromosome 12q23.1 and is transcribed from the antisense strand in the opposite direction of *TMPO* and composed of 2 exons (Supplementary Fig. 1a). To check the coding potential, we performed the in silico analysis with the Coding Potential Assessment Tool (CPAT) to calculate the score for *TMPO-AS1*. According to CPAT analysis, the coding probability of *TMPO-AS1* is 0.001, which is lower than that of other well-characterized lncRNAs, such as *nuclear paraspeckle assembly transcript 1 (NEAT1), colon cancer-associated transcript 1 (CCAT1), and NF-κB interacting lncRNA (NKILA)* (Supplementary Fig. 1b). In addition, for in vitro validation of the peptide-coding potential, the *TMPO-AS1* sequence was inserted upstream of 3× Flag-Tag cassette in a plasmid, transfected into HEK293T cells, and immunoblotted with the Flag antibody. Consistent with the very low coding probability calculated by CPAT, no peptide or protein was detected (Supplementary Fig. 1c).

### *TMPO-AS1* expression is upregulated in ESCC and associated with poor prognosis in patients

Analysis of TCGA data showed upregulated *TMPO-AS1* expression in tumor samples compared to normal tissues in various types of cancer tissues (Supplementary Fig. 1d), especially in ESCA tissues (Fig. 1c). In addition, survival analysis showed that patients with high *TMPO-AS1* expression had shorter overall survival (OS) times across the whole set of various types of cancers (Supplementary Fig. 1e), suggesting that *TMPO-AS1* may be a pancancer oncogene. Specifically, high *TMPO-AS1* expression was also correlated with an unfavorable outcome in TCGA-ESCA patients (Supplementary Fig. 1f, *n* = 74). Because ESCC is one of the most predominant subtypes of ESCA, we verified that the *TMPO-AS1* expression level was significantly higher in ESCC tissues (Fig. 1d). We also performed survival analysis in our independent ESCC cohort (Sun Yat-sen University Cancer Center (SYSUCC), *n* = 108). We categorized the *TMPO-AS1* expression level according to the median value: the expression level was defined as high if higher than the median value and as low otherwise. High *TMPO-AS1* expression was associated with unfavorable OS in patients with ESCC (Fig. 1e). The clinical characteristics of this cohort are shown in Supplementary Table 5. In addition, multivariate analysis showed that *TMPO-AS1* was an independent prognostic factor in patients with ESCC (Supplementary Table 6).

Then, we examined the distribution of *TMPO-AS1* by performing fluorescence in situ hybridization (FISH) and subcellular fractionation assays followed by qPCR. Our results showed that *TMPO-AS1* was localized predominantly in the nucleus, with a small amount localized in the cytoplasm, similar to the distribution pattern of the well-characterized nuclear lncRNA *U6* (Fig. 1f, g, Supplementary Fig. 1g).

Next, we examined *TMPO-AS1* expression in a panel of ESCC cell lines and two normal esophageal epithelial cell lines (Het1A and NE1) and found that the *TMPO-AS1* level was significantly higher in the tumor cell lines than in normal cell lines (Supplementary Fig. 1h). We further determined the copy number of *TMPO-AS1* and found that it was also increased in the ESCC cell lines compared to the normal cell lines (Fig. 1h). Together, these findings suggest that *TMPO-AS1* upregulation might play a role in ESCC development.

### *TMPO-AS1* promotes cell proliferation, migration, and invasion in vitro

**Fig. 2:**
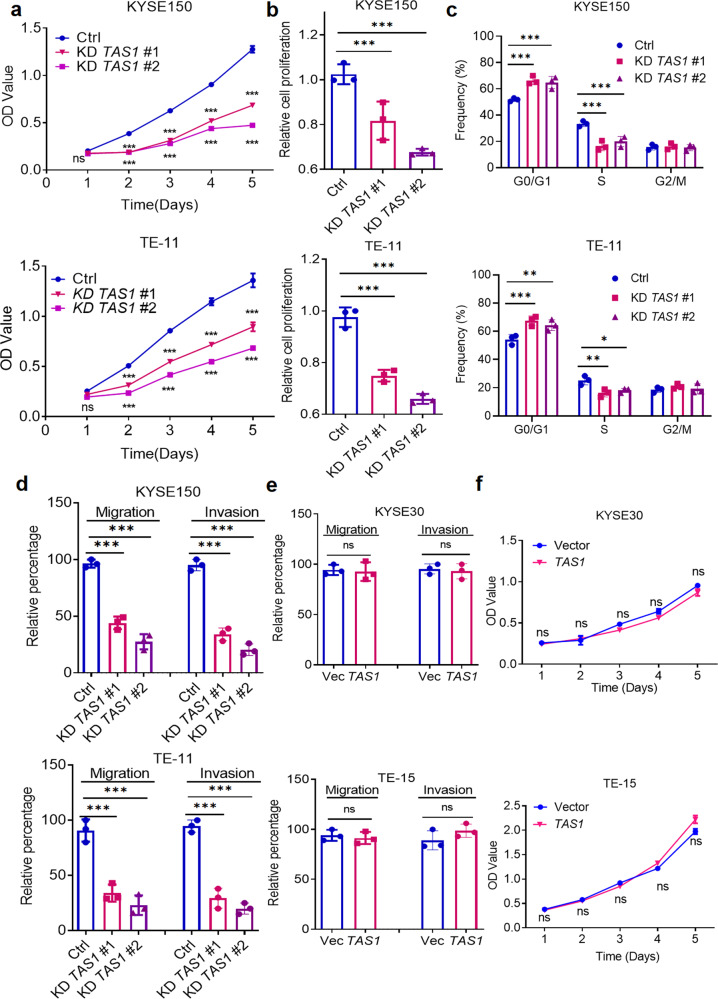
TAS1 promotes cell proliferation, migration and invasion in vitro. **a** MTS assays were performed to measure the proliferation (OD 490 nm) of KYSE150 and TE-11 cells with *TAS1* knockdown (KD) compared with control cells (*n* = 3). **b** BrdU incorporation assays (OD 450 nm) of KYSE150 and TE-11 cells with *TAS1* KD compared with control cells (*n* = 3). **c** Statistical analysis of the cell cycle distribution (%) of KYSE150 and TE-11 cells with *TAS1* KD compared with control cells. **d** Statistical analysis of the migration and invasion rates (%) of KYSE150 and TE-11 cells with *TAS1* KD (*n* = 3). **e** Statistical analysis of the migration and invasion rates (%) of KYSE30 and TE-15 cells with *TAS1* overexpression (OE) (*n* = 3). **f** MTS assays were performed to measure the proliferation of KYSE30 and TE-15 cells with *TAS1* OE compared with control cells (*n* = 3). The data are presented as the mean±S.D. values. *P < 0.05; **P < 0.01; ***P < 0.001; ns, not significant.

We further investigated the oncogenic function of *TMPO-AS1* by customized antisense oligonucleotide (ASO)-induced knockdown and lentiviral-mediated overexpression of *TMPO-AS1* in ESCC cells (Supplementary Fig. 2a–c). The target sequences are shown in Supplementary Table 7. Then, we performed MTS assays and found that *TMPO-AS1* knockdown significantly reduced cell proliferation (Fig. 2a). In addition, BrdU incorporation assays revealed that silencing *TMPO-AS1* reduced ESCC cell proliferation (Fig. 2b). Cell cycle analysis showed that *TMPO-AS1* knockdown resulted in G1/S arrest (Fig. 2c). Furthermore, Transwell assays showed that *TMPO-AS1* silencing inhibited the migration and invasion of ESCC cells (Fig. 2d, Supplementary Fig. 2d). Interestingly, ectopic overexpression of *TMPO-AS1* had minimal effects on these parameters (Fig. 2e, f, Supplementary Fig. 2e).

Consistent with the effects of *TMPO-AS1* on ESCC cell proliferation and migration, we also observed a positive yet non-significant association between *TMPO-AS1* expression and ESCA pathological stage in the TCGA database (Supplementary Fig. 2f).

### *TMPO-AS1* facilitates ESCC tumor growth and metastasis in vivo

**Fig. 3:**
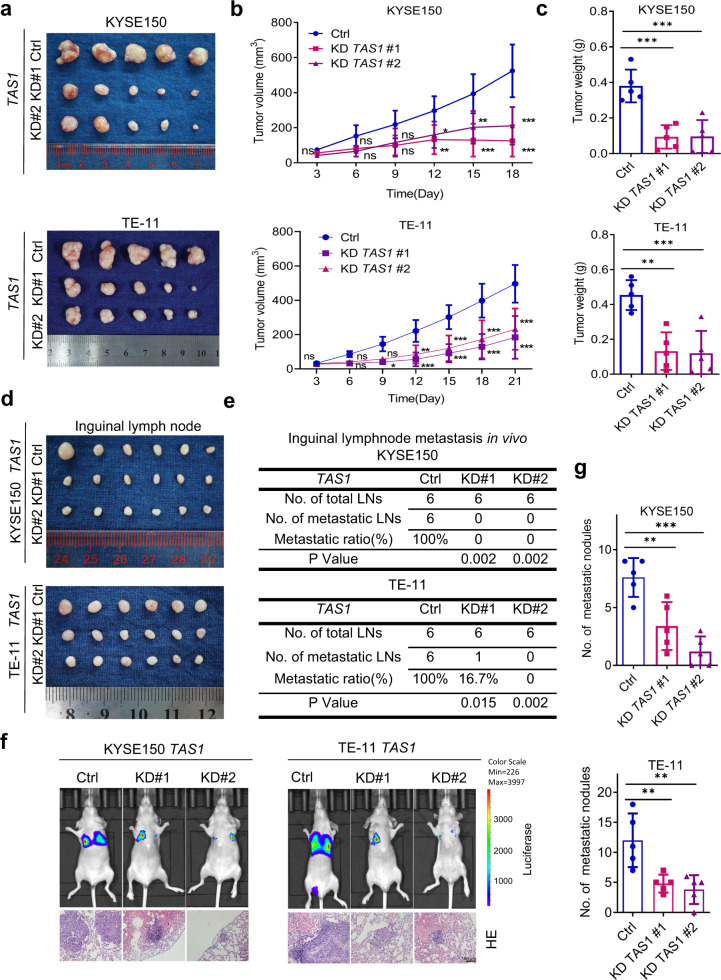
TAS1 facilitates tumor growth and metastasis in vivo. **a** Image of subcutaneous xenograft tumors formed by KYSE150 and TE-11 cells transduced with sh*TAS1* #1, sh*TAS1* #2 or shCtrl in nude mice. (*n* = 5). **b**, **c** Subcutaneous tumor volume curve and statistical analysis of the weight of tumors formed by KYSE150 and TE-11 cells treated as indicated (*n* = 5). **d** Image of popliteal lymph nodes harvested 8 weeks after injection of KYSE150 and TE-11 cells with lentiviral shRNA vector-mediated *TAS1* KD into the left footpads of nude mice (*n* = 6). **e** Statistical analysis of the incidence of popliteal lymph node metastasis in the indicated groups (chi-square test, two-sided). **f** Representative images of whole-body in vivo bioluminescence and H&E staining (scale bar, 100 μm) in lung sections from mice injected via the tail vein with KYSE150 and TE-11 cells with stable *TAS1* (#1 and #2) knockdown or control (Ctrl) cells on Day 56 postinjection. g. Statistical analysis of the metastatic lung nodules confirmed by H&E staining (*n* = 5). The data are presented as the mean±S.D. values. *P < 0.05; **P < 0.01; ***P < 0.001; ns, not significant.

Next, we explored the role of *TMPO-AS1* in tumorigenesis and tumor development in vivo. In the subcutaneous cell line-derived xenograft (CDX) model, *TMPO-AS1* knockdown significantly inhibited tumor growth, as indicated by the decreased tumor volume and tumor weight (Fig. 3a–c). Then, we established a popliteal sentinel lymph node metastasis model in nude mice to evaluate the effects of *TMPO-AS1* on ESCC lymph node metastasis^15^. The popliteal lymph nodes were harvested 8 weeks after tumor cell injection (Fig. 3d). The lymph nodes weighed slightly less in the *TMPO-AS1* knockdown group than in the control group (Supplementary Fig. 3a). The metastasis-positive lymph nodes were identified by examining H&E-stained serial sections of each inguinal lymph node for metastatic micronodules. At least one locus of metastatic micronodules was required for classification as a metastasis-positive lymph node. Representative pictures of metastatic micronodules are shown and marked in Supplementary Fig. 3b. Our data revealed a significantly reduced metastasis ratio in the *TMPO-AS1*-silenced group (Fig. 3e), suggesting that *TMPO-AS1* knockdown suppressed lymph node metastasis of ESCC. In addition, tail vein injection of *TMPO-AS1*-knockdown cells or control cells was performed to examine lung metastasis. In vivo bioluminescence imaging showed a decreased luminescence intensity in the lungs of mice injected with cells group compared to control cells (Fig. 3f). H&E staining of serial sections of lung tissues was performed to confirm metastasis and quantify metastatic nodules (Fig. 3f). The results showed significantly reduced numbers and volumes of metastatic nodules in the *TMPO-AS1*-silenced group (Fig. 3g), indicating that *TMPO-AS1* knockdown suppressed hematogenous metastasis of ESCC.

### *TMPO-AS1* performs its biological functions by regulating *TMPO* in ESCC

**Fig. 4:**
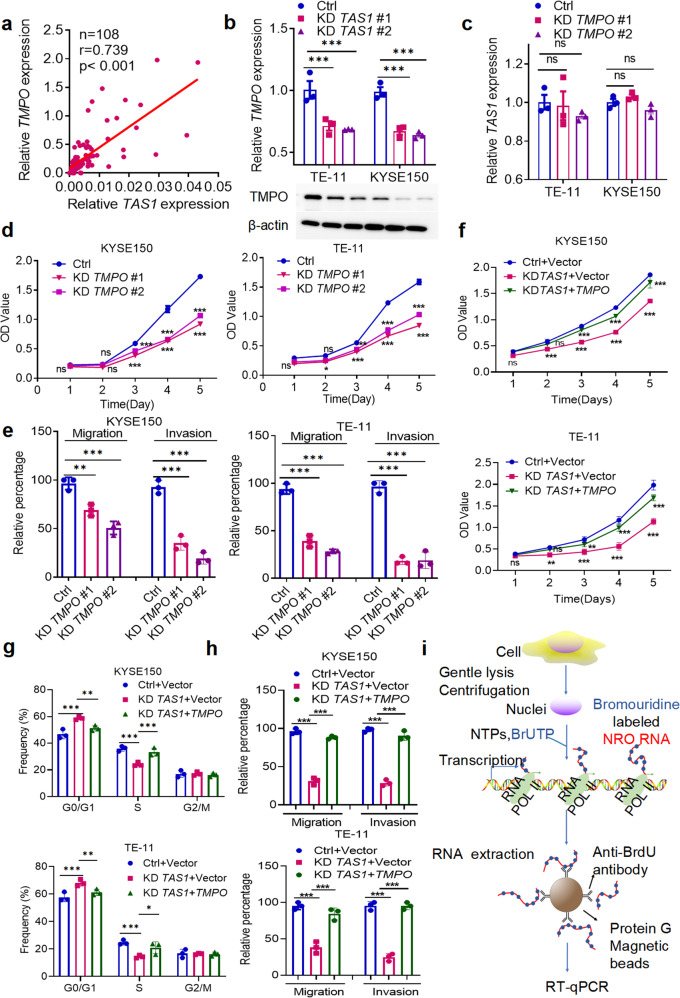
TAS1 performs its biological functions by *cis*-activating TMPO transcription. **a** The correlation between *TAS1* and *TMPO* mRNA expression in clinical ESCC tissues (SYSUCC, *n* = 97, Pearson correlation analysis). **b** Detection of *TMPO* expression by qPCR and WB in KYSE150 and TE-11 cells with *TAS1* KD compared with control cells (*n* = 3). **c** Detection of *TAS1* expression by qPCR in KYSE150 and TE-11 cells with *TMPO* KD compared with control cells (*n* = 3). **d** MTS assays were performed to evaluate the proliferation (OD 490 nm) of KYSE150 and TE-11 cells with *TMPO* KD (*n* = 3). **e** Statistical analysis of the migration and invasion rates (%) of KYSE150 and TE-11 cells with *TMPO* KD (*n* = 3). **f**–**h** MTS assays and statistical analysis of the cell cycle distribution (%) of KYSE150 cells treated as indicated and the migration and invasion rates (*n* = 3). **i** A schematic diagram of the NRO assay. The data are presented as the mean±S.D. values. *P < 0.05; **P < 0.01; ***P < 0.001; ns, not significant.

*TMPO* is located on the opposite strand of *TMPO-AS1* on chromosome 12q21.2 and is the cognate gene of *TMPO-AS1*. Evidence suggests that *TMPO* plays diverse roles in various cancers^18–21^. Since some antisense lncRNAs perform their biological functions by regulating neighboring genes^12,13,22^, we investigated the regulatory relationship between *TMPO* and *TMPO-AS1* expression in ESCC tissues. We found that *TMPO* expression was positively correlated with *TMPO-AS1* expression in the SYSUCC-ESCC dataset (Fig. 4a). Furthermore, *TMPO-AS1* silencing obviously reduced the expression of *TMPO* (Fig. 4b), whereas ectopic overexpression of *TMPO-AS1* did not affect the *TMPO* level (Supplementary Fig. 4a). In contrast, *TMPO* silencing had no effect on *TMPO-AS1* expression (Fig. 4c). The ASOs and siRNAs were designed to specifically target the nonoverlapping sequences of these two genes to exclude any off-target effects. Specific silencing of *TMPO* was confirmed by qPCR and WB analyses (Supplementary Fig. 4b).

Similar to the *TMPO-AS1* expression pattern in ESCC, the TMPO expression level was also increased in ESCC tissues, as confirmed by qPCR and immunohistochemistry (IHC) (Supplementary Fig. 4c, d). TMPO was also upregulated in most ESCC cells (Supplementary Fig. 4e). We next investigated the role of *TMPO* in ESCC. Consistent with the phenotypes we observed after *TMPO-AS1* knockdown, the MTS assay showed that *TMPO* silencing reduced ESCC cell proliferation (Fig. 4d). Cell cycle analysis revealed induction of G1/S phase arrest after *TMPO* knockdown (Supplementary Fig. 4f). Transwell assays revealed that *TMPO* knockdown inhibited ESCC cell migration and invasion (Fig. 4e, Supplementary Fig. 4g). Thus, *TMPO* promotes cell proliferation, migration and invasion, mimicking the effects of *TMPO-AS1*, on ESCC cells.

We conducted a series of rescue experiments to investigate whether *TMPO-AS1* performs its function in ESCC by regulating *TMPO*. Consistent with our prediction, MTS and Transwell assays showed that *TMPO* overexpression in *TMPO-AS1*-silenced cells decreased the inhibition of cell proliferation, G1/S progression, migration and invasion (Fig. 4f-h, Supplementary Fig. 4h). Collectively, these data suggest that *TMPO-AS1* might promote ESCC tumorigenesis and metastasis by regulating *TMPO* expression.

### *TMPO-AS1* regulates the transcription of its cognate sense gene *TMPO in cis*

Numerous antisense lncRNAs have been reported to regulate the transcription of their cognate genes^12,13^. *TMPO-AS1* is a NAT lncRNA transcribed in the opposite direction starting from the first intron in the antisense strand of *TMPO*, and it includes the transcription start site (TSS) and the 5’UTR of *TMPO* (Supplementary Fig. 1a). Therefore, we conducted an NRO assay to evaluate the regulation between *TMPO-AS1* and *TMPO*. NRO assays can measure the transcription efficiency without the influence of degradation by labeling nascent transcripts with bromouridine (Fig. 4i). The results showed that *TMPO-AS1* knockdown reduced the level of nascent *TMPO* mRNA transcripts (Supplementary Fig. 5a, b). We also evaluated *TMPO* mRNA stability and found that *TMPO-AS1* did not affect the degradation rate of *TMPO* mRNA in the presence of the transcription inhibitor actinomycin D (ActD) (Supplementary Fig. 5c). Together, these results suggest that *TMPO-AS1* regulates *TMPO* transcription instead of affecting *TMPO* mRNA stability. Combined with the observation that ectopic expression of *TMPO-AS1* exerted minimal effects, our results indicate that *TMPO-AS1* might act *in cis* but not in trans to activate *TMPO* expression.

### *TMPO-AS1* increases the H3K27ac level in the *TMPO* promoter by recruiting FUS and p300 proteins to form biomolecular condensates

**Fig. 5:**
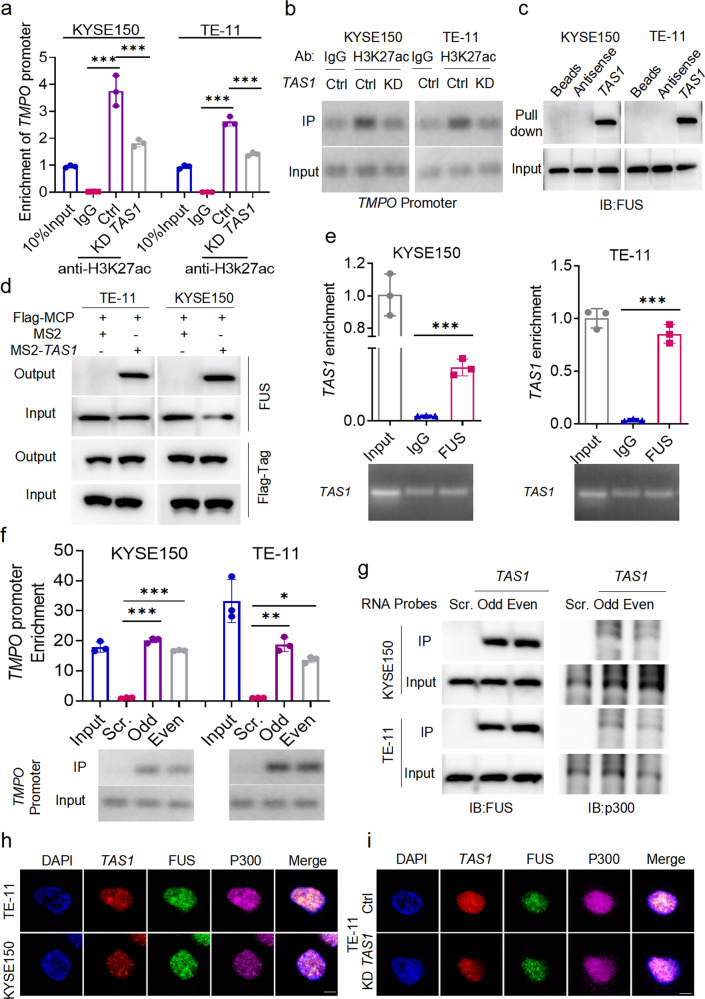
TAS1 regulates H3K27ac enrichment in the TMPO promoter by recruiting FUS and p300 to form condensates. **a**, **b** Enrichment of the *TMPO* promoter by ChIP using an anti-H3K27ac antibody in KYSE150 and TE-11 cells with or without *TAS1* KD were evaluated. The *TMPO* promoter level in the 10% input sample is set to 1. Primer locations in the *TMPO* promoter are shown at the bottom of Supplementary Fig. 5d. The primer set P3 was used to obtain the results shown (*n* = 3). **c** FUS in cell lysates was pulled down by biotin-labeled *TAS1* but not its antisense RNA. **d** *TAS1* binding proteins were detected using MS2-TRAP and WB analysis. *TAS1*-bound FUS was captured on anti-Flag antibody-conjugated affinity agarose beads; IP complexes were separated and identified using specific antibodies. **e** RIP assays indicated that *TAS1* in ESCC cell lysates was enriched by FUS-specific antibodies. **f**, **g** ChIRP-purified DNA and proteins were analyzed using qPCR and western blotting, respectively. Odd, Even and Scr. denote the odd- and even-ranked corresponding probes for *TAS1* and the negative control probes provided by RiboBio. The *TMPO* promoter region represented by P3 was enriched by the *TAS1* probes. FUS and p300 proteins were also precipitated by the *TAS1* probes in ESCC cells. The locations of the primers in the *TMPO* promoter are shown at the bottom of Supplementary Fig. 5d. **h** IF and FISH assays showed that *TAS1*, FUS and p300 were colocalized mostly in the nucleus and existed as puncta. Scale bar: 5 μm. **i** IF and FISH assays showed a reduction in the number of colocalized puncta formed by *TAS1*, FUS and p300 after TAS1 silencing in TE-11 cells. Scale bar: 5 μm. The data are presented as the mean±S.D. values. *P < 0.05; **P < 0.01; ***P < 0.001; ns, not significant.

Next, we examined the gene loci of *TMPO* and *TMPO-AS1* in the UCSC Genome Browser. We found that in different cell types, H3K27ac, which is the hallmark of open chromatin with active transcription, was enriched in the TSS-harboring regions of both genes (Supplementary Fig. 5d). Next, we performed ChIP–qPCR using the anti-H3K27ac antibody in KYSE150 and TE-11 cells. Four pairs of primers (P1-P4) specific for the *TMPO* promoter region were designed, and their sequences are shown at the bottom of Supplementary Fig. 5d. The results of qPCR analysis using P3 revealed that the *TMPO* promoter region was enriched by the anti-H3K27ac antibody (Fig. 5a, b). Furthermore, *TMPO-AS1* silencing significantly reduced the H3K27ac level in the *TMPO* promoter region (Fig. 5a, b). Therefore, H3K27ac enrichment in the promoter region might be the reason for the upregulated expression of *TMPO* in ESCC cells.

The molecular function of lncRNAs is closely associated with their subcellular localization^23^. We already determined that *TMPO-AS1* was localized predominantly in the nucleus (Fig. 1f, g, Supplementary Fig. 1f). Nuclear lncRNAs have been reported to recruit chromatin-remodeling proteins to chromatin and thereby control transcriptional activity. NAT lncRNAs also perform their functions by interacting with RNA binding proteins (RBPs). To identify possible *TMPO-AS1*-interacting proteins, we performed a targeted screen of intranuclear RBPs and found that *TMPO-AS1* was very likely to interact with the RBP FUS, with a probability of 0.9 (http://pridb.gdcb.iastate.edu/RPISeq/). FUS is a well-characterized RNA binding protein with various roles in different cellular processes, such as transcriptional regulation, RNA splicing, RNA transport, DNA repair and the DNA damage response^24^. FUS is able to phase separate and form biomolecular condensates with itself or other molecular partners, which drives aberrant chromatin looping and cancer development^25^. Then, we performed RNA pulldown followed by immunoblot analysis on ESCC cell lysates. The results validated the interaction between *TMPO-AS1* and FUS (Fig. 5c). We also performed MS2-tagged RNA affinity purification (MS2-TRAP) and immunoblot analysis to further characterize the interaction between *TMPO-AS1* and *TMPO* in situ. Coexpression of MS2-*TMPO-AS1* and Flag-tag MS2 coat protein (MCP) led to significant enrichment of FUS by the anti-Flag antibody compared with the isotype control, indicating that FUS specifically binds to *TMPO-AS1* (Fig. 5d). This observation was further confirmed by a RIP assay, where *TMPO-AS1* was successfully enriched by the anti-FUS antibody (Fig. 5e). However, FUS expression did not change after *TMPO-AS1* knockdown (Supplementary Fig. 5e). Next, we performed a ChIRP assay, which is based on affinity capture of a target lncRNA-chromatin complex, with biotinylated ASO probes for *TMPO-AS1* and subjected the precipitated products to qPCR and immunoblot analysis; the results indicated that *TMPO-AS1* indeed bound to the promoter sequence of *TMPO* (Fig. 5f), and the immunoblot analysis further confirmed the direct binding between *TMPO-AS1* and FUS (Fig. 5g). Taken together, these results indicate that the expression level of *TMPO-AS1* does not affect the expression level of FUS in ESCC cells but influences FUS recruitment to the *TMPO* promoter.

FUS can form ribonucleoprotein complexes with lncRNAs and recruit the histone acetyltransferase complex to the TSS of target genes to regulate their transcription by interacting with HAT complex members, including p300, CBP, and TIP60^26,27^. Therefore, we performed co-IP with both anti-FUS and anti-p300 antibodies in ESCC cells. We first confirmed the direction of the interaction between FUS and p300 (Supplementary Fig. 5f). Furthermore, ChIRP followed by immunoblotting showed that p300 was enriched in the *TMPO-AS1* probe group compared to the scrambled probe group (Fig. 5g). IF and FISH colocalization analyses showed that *TMPO-AS1*, FUS and p300 were colocalized in the nucleus, and they were observed as puncta, suggesting the formation of lncRNA-protein biomolecular condensates (Fig. 5h). Interestingly, *TMPO-AS1* silencing evidently reduced the number of colocalized puncta (Fig. 5i), indicating that *TMPO-AS1* is likely to facilitate the formation of biomolecular condensates with FUS and p300.

We intended to further identify the downstream factors of *TMPO-AS1* and *TMPO* involved in ESCC progression. A qPCR array containing 12 genes associated with G1/S phase transition and 89 metastasis-related gene probes^28^ (Supplementary Table 8) was used to compare the mRNA expression profiles between *TMPO-AS1*-knockdown cells and control cells as well as between *TMPO*-knockdown cells and control cells as an approach to further identify downstream factors of *TMPO-AS1* and *TMPO* involved in ESCC cell proliferation and metastasis. Interestingly, the expression of CyclinD1 and MTA1 was downregulated after knockdown of either *TMPO-AS1* or *TMPO* (Supplementary Fig. 5g). Immunoblot analysis showed reduced expression of CyclinD1 and MTA1 in *TMPO-AS1*-silenced cells (Supplementary Fig. 5h). Rescue experiments indicated that the downregulation of CyclinD1 and MTA1 expression induced by *TMPO-AS1* silencing was reversed by *TMPO* overexpression (Supplementary Fig. 5i). Collectively, these results reveal that *TMPO-AS1* recruits FUS/p300 to the *TMPO* promoter and forms biomolecular condensates by direct binding, promoting H3K27ac and facilitating the transcription of *TMPO*, resulting in subsequent upregulation of CyclinD1 and MTA1, ultimately leading to ESCC tumor development.

### Effects of *TMPO-AS1* targeting on ESCC tumors in vivo

**Fig. 6:**
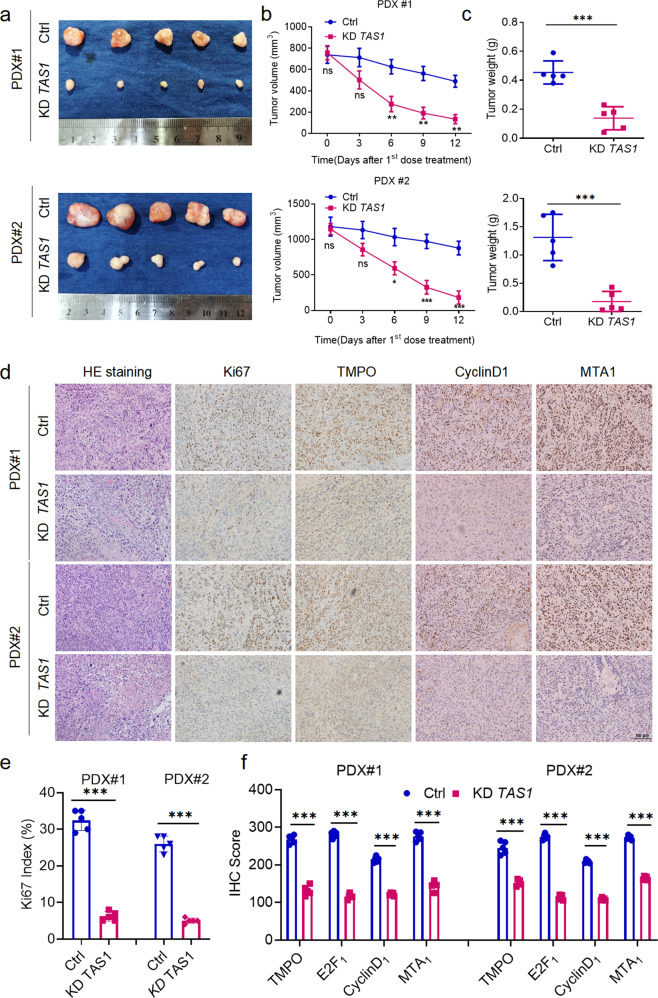
TAS1 constitutes a potential therapeutic target in ESCC. **a** Images of ex vivo tumors from the ESCC PDX model (*n* = 5). **b**, **c** Tumor volume curve and statistical analysis of the tumor weight of the PDX tumors. **d** Representative images of H&E staining and immunohistochemical staining for Ki67, TMPO, CyclinD1 and MTA1 in randomly selected PDX tumors from each group. Scale bar, 100 μm. **e** Statistical analysis of the Ki67 proliferation index (*n* = 5). **f** Statistical analysis of the immunohistochemical scores for the indicated genes (*n* = 5). The data are presented as the mean±S.D. values. *P < 0.05; **P < 0.01; ***P < 0.001; ns, not significant.

To examine the therapeutic potential of targeting *TMPO-AS1*, we established PDX models derived from two patients diagnosed with ESCC at SYSUCC. We injected ASOs against *TMPO-AS1* optimized in the in vitro study intratumorally into PDX-bearing BALB/c nude mice, which resulted in marked decreases in the tumor volume and tumor weight (Fig. 6a–c), suggesting the promising therapeutic potential of targeting *TMPO-AS1*. H&E staining of the excised tumors showed no obvious morphological differences between the treatment group and the control group (Fig. 6d). Immunohistochemical staining showed that *TMPO-AS1* knockdown significantly impaired tumor proliferation, as indicated by the reduced Ki67 index (Fig. 6d, e). Accordingly, the expression levels of TMPO and the downstream proteins CyclinD1 and MTA1 were also obviously reduced, consistent with the results described above (Fig. 6d, f).

### The *TMPO-AS1*/*TMPO* axis is associated with ESCC development

**Fig. 7:**
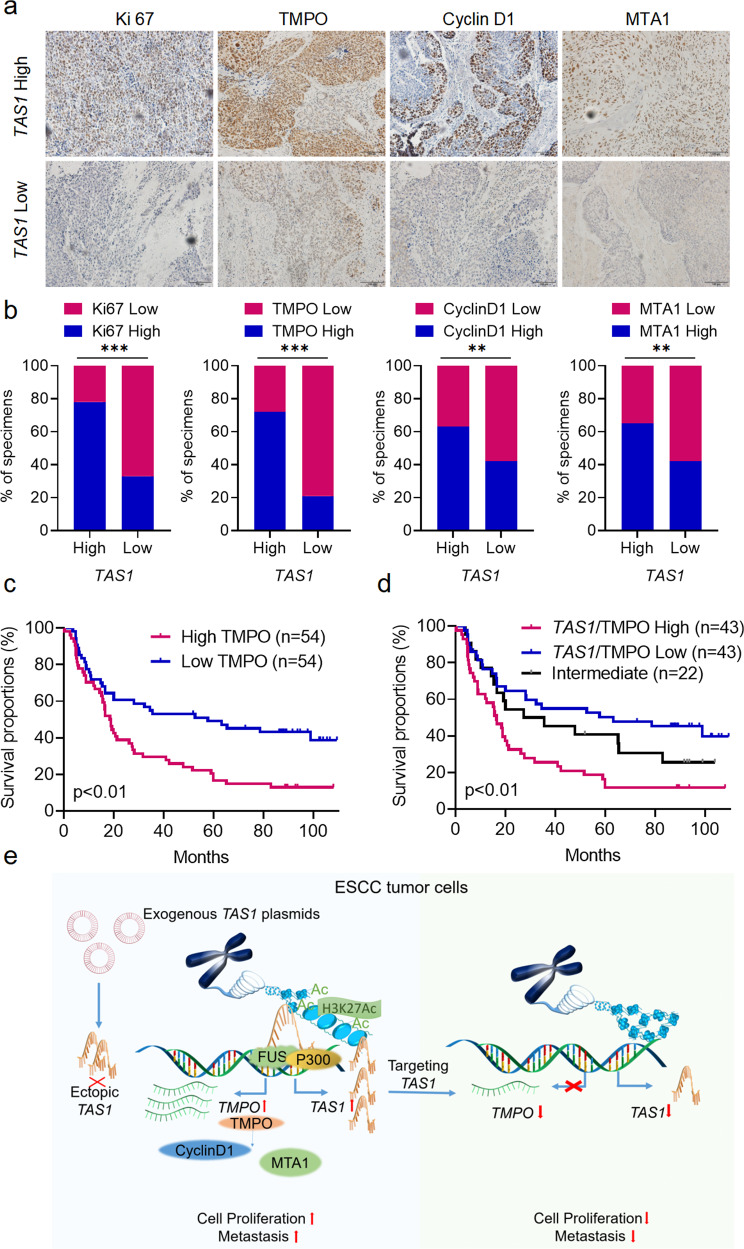
Clinical relevance of the TAS1/TMPO axis in ESCC. **a** Representative images of immunohistochemical staining for Ki67, TMPO, CyclinD1 and MTA1 in tissues from patients with ESCC exhibiting low or high *TAS1* expression. Scale bar, 100 µm. **b** Percentage of specimens with low or high Ki67, TMPO, CyclinD1 and MTA1 expression in the low and high *TAS1* expression groups (SYSUCC, *n* = 108, chi-square test, two-sided). **c** Kaplan–Meier analysis of OS for patients with ESCC (SYSUCC) exhibiting low (*n* = 54) or high (*n* = 54) TMPO expression (log-rank test, two-sided). **d** Kaplan–Meier analysis of OS for patients with ESCC (SYSUCC) exhibiting low (low expression of both *TAS1* and TMPO, *n* = 43), high (high expression of both *TAS1* and TMPO, *n* = 43) or intermediate (*n* = 22) *TAS1*/TMPO expression (log-rank test, two-sided). **e** Graphical abstract showing that the lncRNA *TAS1* activates TMPO transcription *in cis* by recruiting FUS and p300 to modulate H3K27ac modification in the promoter region and that targeting *TAS1* attenuates ESCC progression. The data are presented as the mean ± S.D. values. *P < 0.05; **P < 0.01; ***P < 0.001; ns, not significant.

We used a cohort of ESCC tissues (SYSUCC, *n* = 108; clinicopathological information is provided in Supplementary Table 9) to analyze *TMPO-AS1* expression using qPCR and to analyze TMPO, Ki67, CyclinD1, and MTA1 expression using IHC in order to collectively evaluate whether the *TMPO-AS1*/*TMPO* axis is clinically and pathologically relevant in ESCC. TMPO, Ki67, CyclinD1 and MTA1 were expressed at higher levels in the *TMPO-AS1*-high group than in the *TMPO-AS1*-low group (Fig. 7a, b), confirming the promoting effects of *TMPO-AS1* on *TMPO* expression and ESCC progression.

Furthermore, we analyzed the clinical relevance of *TMPO* to patient outcomes. The correlations between TMPO expression and clinicopathological features are shown in Supplementary Table 10. Kaplan–Meier analysis showed that high TMPO expression was associated with poor outcomes in patients with ESCC (Fig. 7c). Then, according to qPCR analysis of *TMPO-AS1* and immunohistochemical staining of TMPO, the samples were classified into the *TMPO-AS1*/TMPO-high, *TMPO-AS1*/TMPO-intermediate, and *TMPO-AS1*/TMPO-low groups, and the patients in the *TMPO-AS1*/TMPO-high subgroup had the worst prognosis among the three groups (Fig. 7d). In summary, these data further indicated that *TMPO-AS1*/TMPO potentially constitute promising prognostic indicators and therapeutic targets in ESCC.

## Discussion

ESCC is a predominant histological subtype of esophageal malignancy, especially in Asia. More than 90% of esophageal cancer cases in the East Asian region are ESCC^29^. With the development of cancer therapies, the survival of patients with ESCC has improved. However, the overall therapeutic effect is poor due to the lack of promising targets, with a 5-year survival rate of less than 10% for patients with advanced disease. Therefore, studies aiming to further elucidate the molecular mechanisms underlying the development of ESCC are urgently needed. Recently, lncRNAs have emerged as important epigenetic regulators that play essential roles in various physiological and pathological processes^30,31^. The functions and mechanisms of lncRNAs have been increasingly appreciated in different cancers^32^. For example, lncRNAs have been reported to be associated with diverse pathological functions, including tumor proliferation, metastasis, angiogenesis, metabolism, and microenvironmental remodeling^12,33,34^. Therefore, we intended to identify functionally essential lncRNAs in ESCC by performing phenotypic screening of aberrantly expressed lncRNAs using a siRNA library based on TCGA transcriptomic data. *TMPO-AS1*, an antisense lncRNA of *TMPO* located on chromosome 12q23.1, was the candidate with the most potent suppressive effects in our screen. *TMPO-AS1* expression was upregulated in ESCC, and high *TMPO-AS1* expression indicated poor prognosis in patients with ESCC (Fig. 1).

Recent studies have reported that various lncRNAs are abnormally expressed and have crucial functions in ESCC. For example, Zhang et al. revealed that the lncRNA *DNM3OS* regulates the DNA damage response, which results in radioresistance during ESCC treatment^9^. A study by Li et al. showed that the long intergenic noncoding RNA *POU3F3* promotes ESCC tumor growth by interacting with EZH2 to increase the methylation of *POU3F3* and reduce POU3F3 expression^35^. *TMPO-AS1* expression has been reported to be upregulated in various cancers, including bladder cancer, pancreatic cancer, and lung adenocarcinoma^36–38^. However, the role of *TMPO-AS1* in ESCC is less understood. In this study, we reported that *TMPO-AS1* promotes tumor progression through activation of *TMPO* transcription *in cis* in ESCC. Functionally, *TMPO-AS1* promoted ESCC cell proliferation and metastasis both in vitro and in vivo (Figs. 2, 3). Mechanistically, *TMPO-AS1* performed its function by activating *TMPO* transcription *in cis* (Figs. 4, 5). *TMPO-AS1* promoted *TMPO* transcription by recruiting FUS and p300 and forming condensates in situ to acetylate lysine 27 of histone 3 in the *TMPO* promoter (Fig. 5). *TMPO*, also termed *lamina-associated polypeptide 2 (LAP2)*, is the cognate neighboring gene of *TMPO-AS1* located on chromosome 12q21.2, and 6 nuclear isoforms can be produced through alternative splicing. Evidence suggests important roles for *TMPO* in various cancers—*TMPO* expression is upregulated in non-small-cell lung cancer^18^, glioblastoma^39^, and digestive tract carcinomas^21^, although little is known about its role in ESCC.

Among the various types of lncRNAs, NAT lncRNAs are attracting increasing attention. NAT lncRNAs are widespread in the genomes of diverse species, including humans^40,41^. These NATs and their cognate genes often show concordant or discordant expression patterns^42^. Diverse transcriptional or posttranscriptional mechanisms have been associated with the ability of NATs to regulate the expression of their sense transcripts. *Cis*-acting NAT lncRNAs serve as scaffolds to recruit chromatin-modulating proteins to facilitate DNA methylation, histone modification, and chromatin remodeling, ultimately leading to activated transcription of the cognate gene. NAT lncRNAs may compete with their sense transcripts for binding of RNA polymerase II (RNA Pol II) and regulatory transcription factors, resulting in transcriptional interference. *Trans*-acting NAT lncRNAs may affect mRNA stability or modulate protein translation.

For the first time, we reported the transcriptional activation of *TMPO* mediated by *TMPO-AS1* (Fig. 7e). Li et al. reported that *TMPO-AS1* promotes thyroid cancer cell proliferation by sponging miR-498 to increase *TMPO* expression^43^. Here, we found that *TMPO-AS1* acts *in cis* to activate *TMPO* expression at the transcriptional level. The difference in the mechanism by which *TMPO-AS1* regulates *TMPO* expression might be tissue specific. The model we proposed echoes the roles played by the lncRNA *SATB homeobox 2 antisense RNA 1* (*SATB2-AS1*) in promoting SATB2 expression^12^, the *lncRNA homeobox A cluster (HOXA) transcript at the distal tip* (*HOTTIP*) in activating *HOXA* gene expression^44^, and the lncRNA *HEAL* in regulating HIV-1 replication^26^. However, the underlying mechanisms employed by these lncRNAs are different. For example, *HOTTIP* interacts with WDR5 and recruits the MLL complex to maintain H3K4me3 and activate *HOXA* gene transcription. However, *HOTTIP* requires chromosome looping to bring the *HOTTIP* locus spatially closer to its target genes for its *cis*-regulatory action^44^. The different mechanisms might be due to differences in the distances between the TSSs of NAT lncRNAs and their cognate genes. As exemplified by *TMPO-AS1*, the expression of some lncRNAs is correlated with that of their sense protein-coding genes (Fig. 4). This finding may reflect the observation that NAT lncRNAs are essential for regulating the expression of their paired genes, suggesting that this *cis*-regulatory mechanism might be universal for NAT lncRNAs.

LncRNAs are attracting increasing attention as novel therapeutic targets, especially in cancer^45^. Treatments targeting lncRNAs have also become feasible due to technological developments^45–47^. For example, some ASO-based therapies have recently been evaluated in clinical trials^48^. With the successful application of RNA-based vaccinations against COVID-19, the prospects of RNA-based therapeutics are promising. The results of in vivo targeted therapy in the PDX model revealed the potential of *TMPO-AS1* as an effective therapeutic target in ESCC (Fig. 6). Our work showed that the expression of both *TMPO-AS1* and *TMPO* was upregulated in ESCC and that high expression of either *TMPO-AS1* or *TMPO* was strongly associated with unfavorable patient outcomes. Furthermore, high expression of both *TMPO-AS1* and *TMPO* was associated with even worse prognosis, suggesting that the combination of both genes might constitute a more potent prognostic marker in patients with ESCC (Figs. 1, 7).

In summary, our current study showed that *TMPO-AS1* expression was upregulated in ESCC and that high *TMPO-AS1* expression was associated with poor prognosis. *TMPO-AS1* promotes ESCC cell proliferation and metastasis by activating *TMPO* transcription *in cis*. These data suggest that *TMPO-AS1* and TMPO may be novel biomarkers and promising diagnostic and therapeutic targets in ESCC. However, further studies must be performed to elucidate the precise molecular mechanisms by which TMPO might regulate cancer cell proliferation and metastasis in ESCC.

## Supplementary information

Supplementary Materials

## Data Availability

All data generated during this study are included in this published article and its supplementary files.

## References

[1] Bray F (2018). Global cancer statistics 2018: GLOBOCAN estimates of incidence and mortality worldwide for 36 cancers in 185 countries. CA Cancer J. Clin..

[2] Chen W (2016). Cancer statistics in China, 2015. CA Cancer J. Clin..

[3] Enzinger PC, Mayer RJ (2003). Esophageal cancer. N. Engl. J. Med..

[4] Cancer Genome Atlas Research Network. (2017). Integrated genomic characterization of oesophageal carcinoma. Nature.

[5] Kopp F, Mendell JT (2018). Functional classification and experimental dissection of long noncoding RNAs. Cell.

[6] Beermann J, Piccoli MT, Viereck J, Thum T (2016). Non-coding RNAs in development and disease: background, mechanisms, and therapeutic approaches. Physiol. Rev..

[7] Tan YT (2020). LncRNA-mediated posttranslational modifications and reprogramming of energy metabolism in cancer. Cancer Commun..

[8] Xiang X (2021). Cellular senescence in hepatocellular carcinoma induced by a long non-coding RNA-encoded peptide PINT87aa by blocking FOXM1-mediated PHB2. Theranostics.

[9] Zhang H (2019). Cancer-associated fibroblast-promoted LncRNA DNM3OS confers radioresistance by regulating DNA damage response in esophageal squamous cell carcinoma. Clin. Cancer Res..

[10] Liu J (2020). Long noncoding RNA AGPG regulates PFKFB3-mediated tumor glycolytic reprogramming. Nat. Commun..

[11] Katayama S (2005). Antisense transcription in the mammalian transcriptome. Science.

[12] Xu M (2019). LncRNA SATB2-AS1 inhibits tumor metastasis and affects the tumor immune cell microenvironment in colorectal cancer by regulating SATB2. Mol. Cancer.

[13] Zhao X (2018). Global identification of Arabidopsis lncRNAs reveals the regulation of MAF4 by a natural antisense RNA. Nat. Commun..

[14] Wu QN (2021). MYC-activated LncRNA MNX1-AS1 promotes the progression of colorectal cancer by stabilizing YB1. Cancer Res..

[15] Ito T (2008). Pituitary tumor-transforming 1 increases cell motility and promotes lymph node metastasis in esophageal squamous cell carcinoma. Cancer Res..

[16] Roberts TC (2015). Quantification of nascent transcription by bromouridine immunocapture nuclear run-on RT-qPCR. Nat. Protoc..

[17] Chu, C., Quinn, J. & Chang, H. Y. Chromatin isolation by RNA purification (ChIRP). *J. Vis. Exp*. 10.3791/3912 (2012). 10.3791/3912 PMC3460573 22472705

[18] Liu C (2019). Prognostic significance and biological function of Lamina-associated polypeptide 2 in non-small-cell lung cancer. Onco Targets Ther..

[19] Parise P (2006). Lap2alpha expression is controlled by E2F and deregulated in various human tumors. Cell Cycle.

[20] Mirza AN (2019). LAP2 proteins chaperone GLI1 movement between the lamina and chromatin to regulate transcription. Cell.

[21] Kim HJ (2012). LAP2 is widely overexpressed in diverse digestive tract cancers and regulates motility of cancer cells. PLoS One.

[22] Yang MH (2019). Nuclear lncRNA HOXD-AS1 suppresses colorectal carcinoma growth and metastasis via inhibiting HOXD3-induced integrin beta3 transcriptional activating and MAPK/AKT signalling. Mol. Cancer.

[23] Winkler L, Dimitrova N (2022). A mechanistic view of long noncoding RNAs in cancer. Wiley Interdiscip. Rev. RNA.

[24] Yamaguchi A, Takanashi K (2016). FUS interacts with nuclear matrix-associated protein SAFB1 as well as Matrin3 to regulate splicing and ligand-mediated transcription. Sci. Rep..

[25] Ahn JH (2021). Phase separation drives aberrant chromatin looping and cancer development. Nature.

[26] Chao TC (2019). The long noncoding RNA HEAL regulates HIV-1 replication through epigenetic regulation of the HIV-1 promoter. MBio.

[27] Wang X (2008). Induced ncRNAs allosterically modify RNA-binding proteins in cis to inhibit transcription. Nature.

[28] Chen ZH (2019). Eukaryotic initiation factor 4A2 promotes experimental metastasis and oxaliplatin resistance in colorectal cancer. J. Exp. Clin. Cancer Res..

[29] Kamangar F, Dores GM, Anderson WF (2006). Patterns of cancer incidence, mortality, and prevalence across five continents: defining priorities to reduce cancer disparities in different geographic regions of the world. J. Clin. Oncol..

[30] Batista PJ, Chang HY (2013). Long noncoding RNAs: cellular address codes in development and disease. Cell.

[31] Harries LW (2012). Long non-coding RNAs and human disease. Biochem. Soc. Trans..

[32] Huarte M (2015). The emerging role of lncRNAs in cancer. Nat. Med..

[33] Wang Y (2019). LncRNA LINRIS stabilizes IGF2BP2 and promotes the aerobic glycolysis in colorectal cancer. Mol. Cancer.

[34] Sang L (2021). Mitochondrial long non-coding RNA GAS5 tunes TCA metabolism in response to nutrient stress. Nat. Metab..

[35] Li W (2014). Increased levels of the long intergenic non-protein coding RNA POU3F3 promote DNA methylation in esophageal squamous cell carcinoma cells. Gastroenterology.

[36] Xue F (2021). Long non-coding RNA TMPO-AS1 serves as a tumor promoter in pancreatic carcinoma by regulating miR-383-5p/SOX11. Oncol. Lett..

[37] Zhang Y (2021). The long non-coding RNA TMPO-AS1 promotes bladder cancer growth and progression via OTUB1-induced E2F1 deubiquitination. Front. Oncol..

[38] Jiang A (2021). Identification and validation of an autophagy-related long non-coding RNA signature as a prognostic biomarker for patients with lung adenocarcinoma. J. Thorac. Dis..

[39] Zhang L (2016). Depletion of thymopoietin inhibits proliferation and induces cell cycle arrest/apoptosis in glioblastoma cells. World J. Surg. Oncol..

[40] Zhang Y, Liu XS, Liu QR, Wei L (2006). Genome-wide in silico identification and analysis of cis natural antisense transcripts (cis-NATs) in ten species. Nucleic Acids Res..

[41] Cheng J (2005). Transcriptional maps of 10 human chromosomes at 5-nucleotide resolution. Science.

[42] Chen J (2005). Genome-wide analysis of coordinate expression and evolution of human cis-encoded sense-antisense transcripts. Trends Genet..

[43] Li Z (2020). TMPO-AS1 promotes cell proliferation of thyroid cancer via sponging miR-498 to modulate TMPO. Cancer Cell Int..

[44] Wang KC (2011). A long noncoding RNA maintains active chromatin to coordinate homeotic gene expression. Nature.

[45] Esposito R (2019). Hacking the cancer genome: profiling therapeutically actionable long non-coding RNAs using CRISPR-Cas9 screening. Cancer Cell.

[46] Watanabe S (2019). In vivo rendezvous of small nucleic acid drugs with charge-matched block catiomers to target cancers. Nat. Commun..

[47] Bester AC (2018). An integrated genome-wide CRISPRa approach to functionalize lncRNAs in drug resistance. Cell.

[48] Adams BD (2017). Targeting noncoding RNAs in disease. J. Clin. Invest..

