# Supplementary material for: LncRNA TMPO-AS1 promotes esophageal squamous cell carcinoma progression by forming biomolecular condensates with FUS and p300 to regulate TMPO transcription
Source: Exp Mol Med. 2022 Jun 27;54(6):834–47. doi: 10.1038/s12276-022-00791-3 (PMC9243820; doi:10.1038/s12276-022-00791-3)
Supplement: Supplementary file 1 — Supplementary Materials [file 12276_2022_791_MOESM1_ESM.pdf]

Supplementary Fig.1

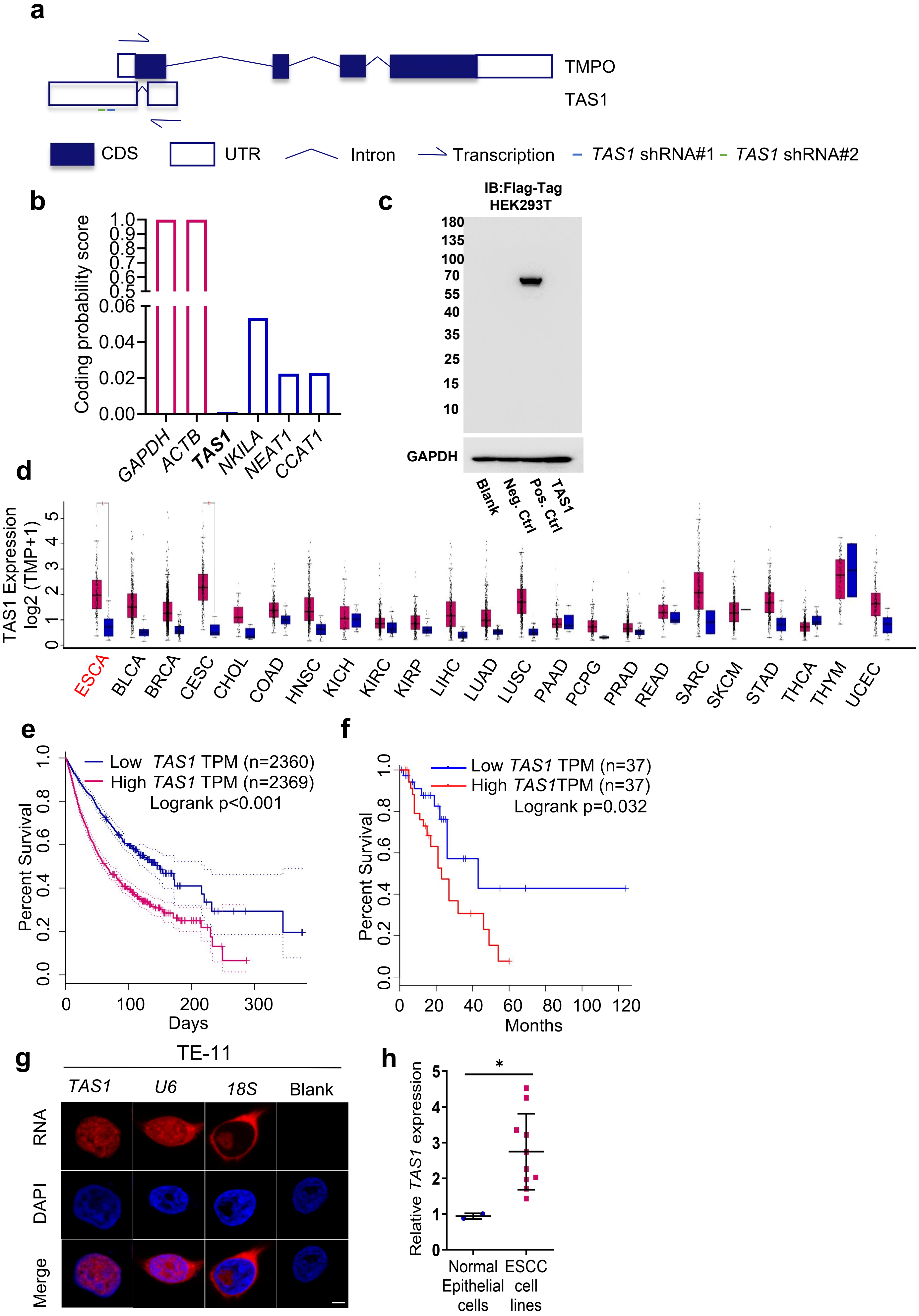

Supplementary Fig.2

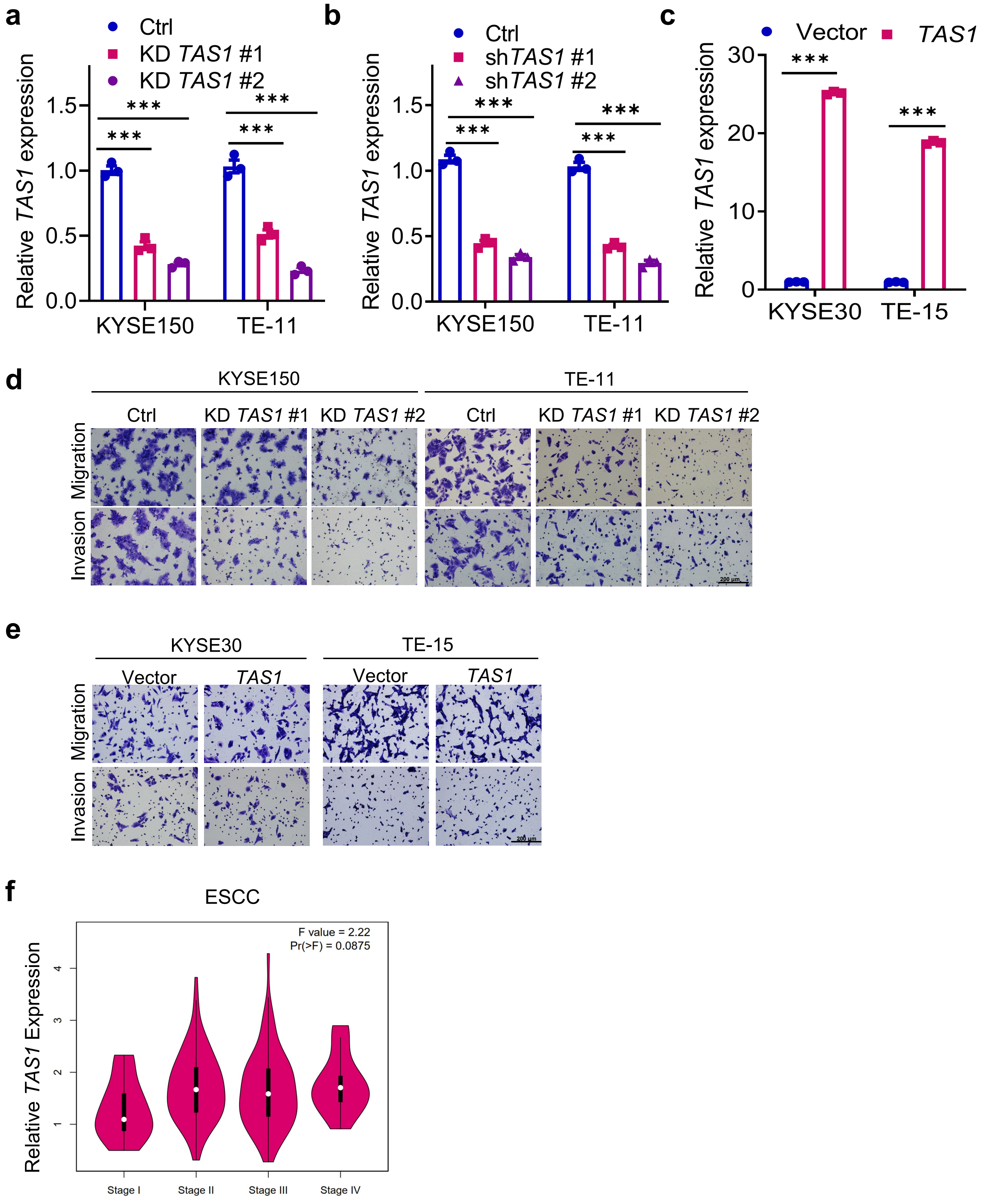

Supplementary Fig.3

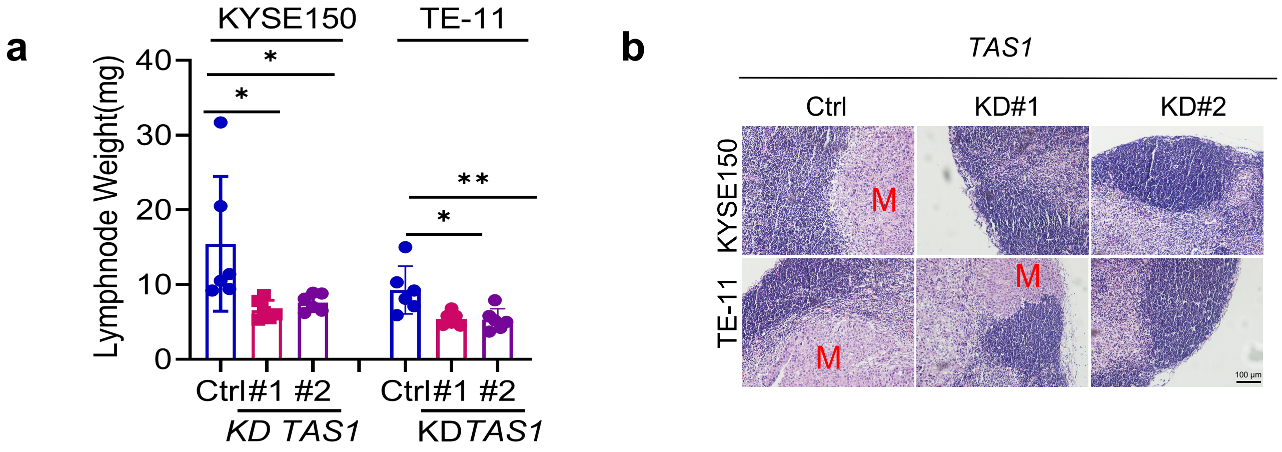

Supplementary Fig.4

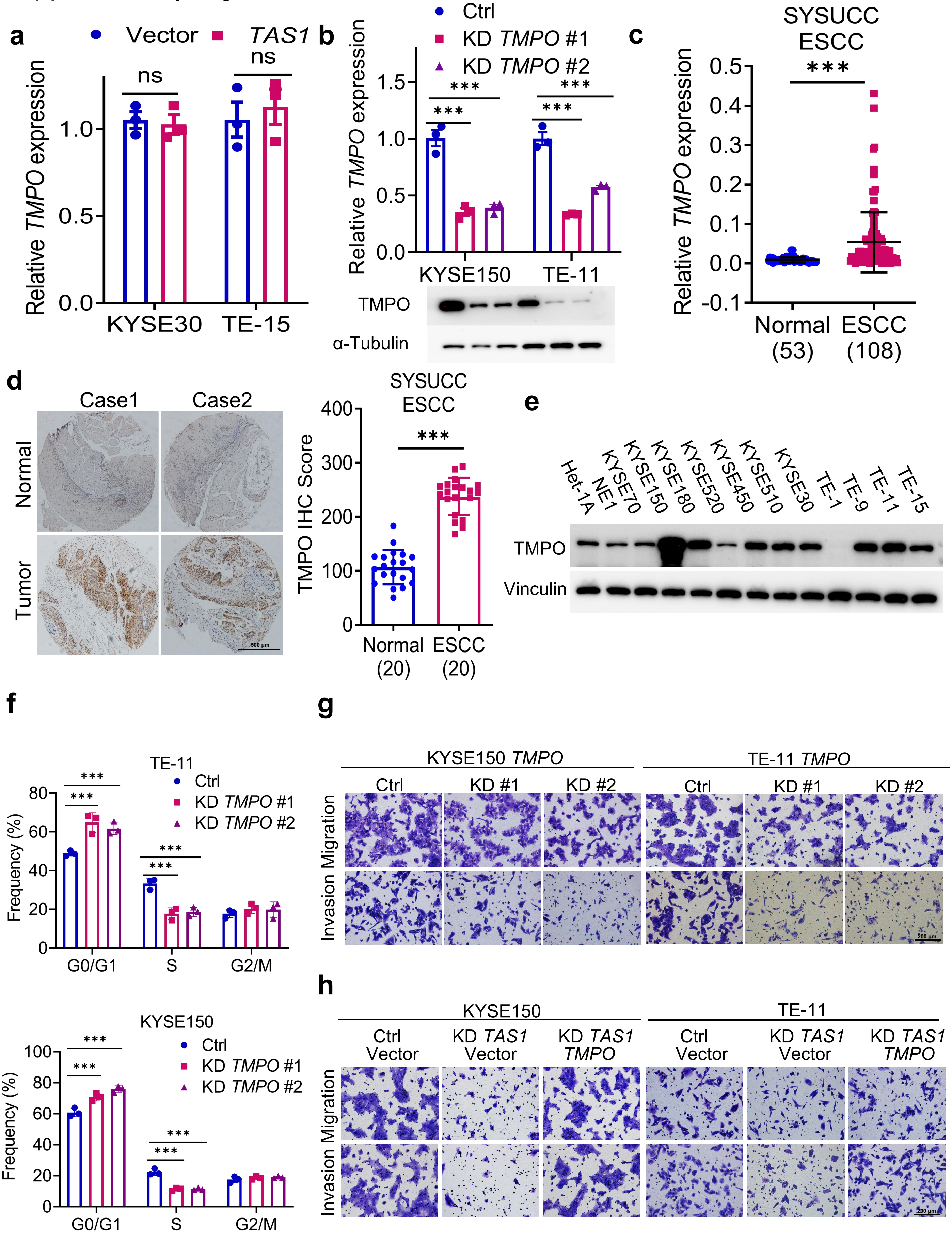

Supplementary Fig.5

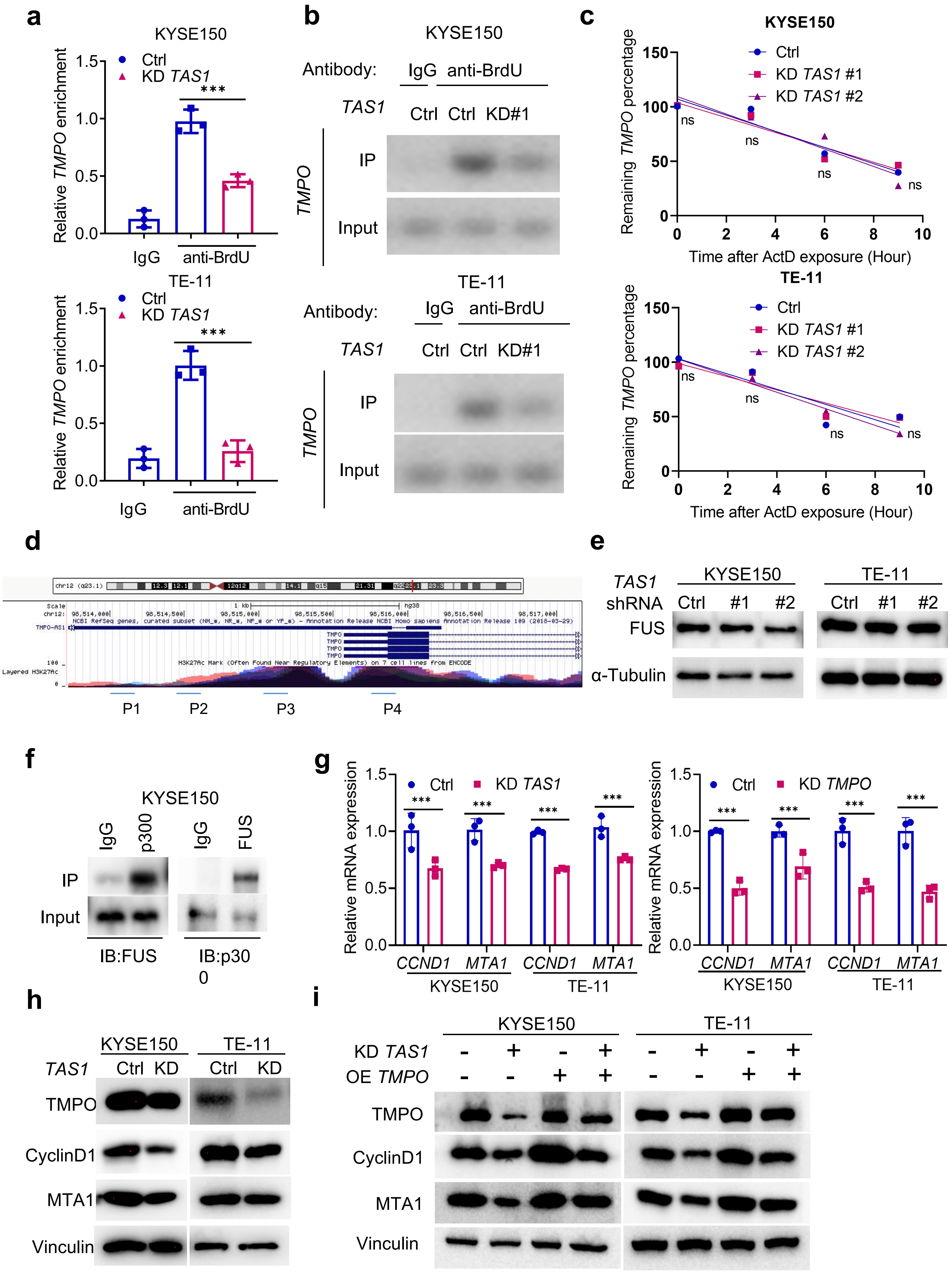

Supplementary Figure legends

Supplementary Fig.1. The lncRNA *TAS1* is upregulated in ESCC and indicates a poor prognosis.

a. Schematic displaying the genomic location of *TAS1* and its cognate gene *TMPO* and the positions of the *TAS1* shRNAs targeting sequences.

b. The coding probabilities of *TAS1* and other lncRNAs assessed using the CPAT; *GAPDH* and *ACTB* served as coding gene references.

c. Immunoblotting results of potential protein or peptide coded by *TAS1*. *TAS1* sequence was cloned upstream of 3xFlag-Tag cassette, transfected in 293 T cells, and immunoblotted for Flag antibody. Positive control was a well characterized coding protein PTBP1 with 3xFlag-Tag.

d. *TAS1* expression in multiple human cancer samples from TCGA data.

e. Kaplan-Meier analysis of the OS curves for a set of patients with multiple cancers presenting with low (n = 2360) or high (n = 2369) *TAS1* expression (log-rank test) from TCGA data.

f. Kaplan-Meier analysis of the OS curves for patients with ESCA presenting low (n = 37) or high (n = 37) *TAS1* expression (log-rank test) from TCGA data.

g. FISH detection of the *TAS1* subcellular localization in TE-11 cells. Scale bar: 5 μm.

h. qPCR detection of *TAS1* expression in 10 ESCC cell lines and 2 normal esophageal epithelial cells.

Data are presented as the means±S.D., *P < 0.05, **P < 0.01, ***P < 0.001, and ns, not significant.

Supplementary Fig.2. *TAS1* promotes cell proliferation, migration and invasion in vitro.

a, b. qPCR detection of *TAS1* knockdown efficiency in KYSE150 and TE-11 cells using ASOs and lentivirus shRNA (n=3).

c. qPCR detection of *TAS1* overexpression efficiency in KYSE30 and TE-15 cells (n=3).

d, e. Transwell assays were used to determine the invasion and migration abilities of KYSE150 and TE-11 cells with *TAS1* KD or KYSE30 and TE-15 cells with *TAS1* OE. Scale bar, 200 μm.

f. Statistical analysis of *TAS1* expression in patients with ESCC of each American Joint Committee on Cancer (AJCC) stage.

Data are presented as the means±S.D., *P < 0.05, **P < 0.01, ***P < 0.001, and ns, not significant.

Supplementary Fig.3. *TAS1* functions as an oncogenic lncRNA in ESCC in vivo.

a. Statistical analysis of the popliteal lymph node weight in the indicated groups (n=6).

b. Representative images of H&E staining of the dissected popliteal lymph nodes from the indicated groups. The metastatic micro nodules were marked. Scale bar, 100 μm.

Data are presented as the means±S.D., *P < 0.05, **P < 0.01, ***P < 0.001, and ns, not significant.

Supplementary Fig.4. *TMPO* is upregulated in ESCC, and *TAS1* exerts its biological functions by cis-activating *TMPO* transcription.

a. qPCR detection of *TMPO* expression in KYSE30 and TE-15 cells with *TAS1* OE compared with the control (n=3).

b. qPCR and WB detection of *TMPO* knockdown efficiency in KYSE150 and TE-11 cells transfected with siRNAs (n=3).

c. qPCR detection of *TMPO* expression in ESCC samples (SYSUCC, n=108).

d. TMPO expression in ESCC tissues measured by IHC and the statistical analysis of IHC score. (SYSUCC, n=20)

e. WB analysis of TMPO expression in a panel of ESCC cell lines(n=10) and two normal esophageal epithelial cells (Het-1A and NE1).

f. Statistical analysis of KYSE150 and TE-11 cells with *TMPO* KD (%) in each cell cycle phase (n=3).

g-h. Transwell assays were used to determine the invasion and migration abilities of KYSE150 and TE-11 cells with indicated treatments. Scale bar, 200 μm.

Data are presented as the means±S.D, *P < 0.05, **P < 0.01, ***P < 0.001, and ns, not significant.

Supplementary Fig.5. *TAS1* regulates H3K27ac enrichment at the *TMPO* promoter by recruiting FUS and p300.

a, b. Nascent *TMPO* mRNA levels were detected using NRO assays followed by qPCR analysis in KYSE150 and TE-11 cells with the indicated treatments.

c. *TMPO* mRNA stability determined by qPCR in KYSE150 and TE-11 cells with the indicated treatments in the presence of ActD.

d. Display of H3K27ac enrichment at the *TMPO* promoter region from the UCSC genome browser data.

e. Immunoblotting of FUS expression after *TAS1* KD in KYSE150 and TE-11 cells.

f. Co-IP analysis confirmed that FUS formed complexes with p300 in KYSE150 cells.

g. A qPCR array was used to screen the downstream target genes of *TAS1*/*TMPO*. CCND1 and MTA1 mRNAs were downregulated after *TAS1*/*TMPO* KD in KYSE150 and TE-11 cells.

h. WB analysis showed that TMPO, CyclinD1 and MTA1 protein levels were reduced after *TAS1* KD.

i. Immunoblotting analysis showed the expression of *TAS1*-regulated genes in KYSE150 and TE-11 cells with *TAS1* knockdown with or without *TMPO* overexpression.

Data are presented as the means±S.D, *P < 0.05, **P < 0.01, ***P < 0.001, and ns, not significant.

Supplementary Methods

RNA isolation and quantitative real-time PCR (qPCR) assays

Total RNA was extracted from cultured cells using an EZ-press RNA Purification Kit (EZBioscience, Shanghai, China) according to instructions. qPCR was performed to determine relative gene expression according to the manufacturer's instructions. The primer sequences used are listed in Supplementary Table 2.

RNA interference and lentivirus transfection

TAS1 antisense oligonucleotides (ASOs) and TMPO siRNAs were provided by RiboBio. Short hairpin RNAs (shRNAs) were provided by OBiO Technology (Shanghai, China). The constructs were verified by sequencing. The sequences are listed in Supplementary Table 10. The position of the targeting sequences were shown in Supplementary Fig. 1 a. KYSE150 and TE-11 cells were transfected with 25 nM ASOs or 50 nM siRNA using Lipofectamine RNAiMAX transfection reagent (Thermo Fisher Scientific, Waltham, MA, USA). KYSE150 and TE-11 cells were infected with lentivirus containing shRNAs targeting *TAS1* in the presence of 5 mg/ml polybrene and selected with 5 μg/ml puromycin (Invivogen, France) to establish stable knockdown cells. Cell transfections and lentiviral infections were performed according to the manufacturer's instructions. Expression efficiency was assessed by qPCR and WB analysis.

Cytosolic/nuclear fractionation

Relative *TAS1* expression in cytoplasmic and nuclear fractions was determined using a Cytoplasmic & Nuclear RNA Purification Kit (Norgen Biotek Corp, Canada) according to the manufacturer's instructions. RNA was extracted from the cytoplasmic and nuclear fractions and subjected to qPCR analysis as described. β-Actin was used as a cytosolic marker, and U6 was used

as a nuclear marker.

Fluorescence in situ hybridization (FISH) assay and immunofluorescence (IF) staining

FISH assays were performed using a lncRNA FISH Kit (RiboBio, Guangzhou, China) according to the manufacturer's instructions. Briefly, ESCC cells were fixed and permeabilized. Next, *TAS1* FISH probes designed by RiboBio were added, and hybridization was carried out overnight in a dark humidified chamber at 37 °C. All images were obtained with a Zeiss LSM880 high-resolution laser confocal microscope (Germany). Cy3 and DAPI channels were used for detection. 18S and U6 were used as markers for the cytosol and nucleus, respectively. IF was performed according to manufacturer's instructions, and anti-FUS antibody (5 μg/ml, Abcam, ab70381) was used.

MS2-tagged RNA affinity purification

ESCC KYSE150 and TE-11 cells were cotransfected with pcDNA3.1-MS2 or pcDNA3.1-MS2-*TAS1* and MCP-3FLAG plasmids (OBiO Technology, Shanghai, China). Forty-eight hours after transfection, living cells were irradiated with 254 nm UV light at 400 mJ per cm^2 for crosslinking. Then, the cell lysates were collected. FLAG-tagged MCP-MS2-*TAS1* was immunoprecipitated with anti-FLAG tag antibody (Cell Signaling Technology, 14793S) and protein A/G magnetic beads (MedChemExpress, HY-K0202). After three washes with low-salt wash buffer, the proteins were eluted with SDS loading buffer and detected by WB analysis.

MTS assay, BrdU cell proliferation assay

ESCC cells were seeded in 96-well culture plates at 800 cells per well. Cell viability was assessed daily for 5 consecutive days using MTS (Qiagen, Hilden, Germany) following the manufacturer's guidelines. The absorbance was measured at a wavelength of 490 nm on a Synergy Multi-Mode

Microplate Reader (Biotek, Vermont, USA). A BrdU cell proliferation assay was performed using a BrdU Cell Proliferation ELISA Kit (Abcam, ab126556) according to the instructions.

Transwell migration assay and Matrigel invasion assay

Migration and invasion assays were performed using 24-well plates and 8-μm pore size Transwell filter inserts (Corning, NY, USA) with or without precoating with Matrigel (Corning, NY, USA). ESCC cells (2×10^5) in FBS-free medium were added to the upper chamber, while the bottom chamber contained medium supplemented with 20% FBS. After incubation at 37 °C for 24 h (migration) or 48 h (invasion), the membrane was washed, fixed and stained with crystal violet (Sangon Biotech, China). Then, invading or migrating cells on the underside of the membrane were counted in five random fields under a microscope.

Western blotting (WB) analysis

WB analysis was performed according to the instructions. Cells were lysed in RIPA lysis buffer. The protein concentrations were calculated using a BCA assay kit (Thermo Fisher Scientific, US). Anti-Vinculin (1:1000, Cell Signaling Technology, 13901), anti-TMPO (1:1000, Affinity Biosciences, DF13264), anti-FUS (1:1000, Abcam, ab70381), anti-p300 (Cell Signaling Technology, 70088S), anti-CyclinD1 (1:1000, Abcam, ab134175), and anti-MTA1 (1:1000, Abcam, ab71153) were used in this study.

Immunohistochemistry (IHC) assays

For the IHC assays, staining and analysis were performed according to the manufacturer's instructions53. Anti-Ki67 (1:250, Abcam, ab15580), anti-LAP2 (1:400, Affinity Biosciences, DF13264), anti-CyclinD1(1:100, Abcam, ab134175), anti-MTA1(1:500, Abcam, ab71153) were used. For quantification analysis, we evaluated the staining area and intensity of all markers.

Cell line-derived xenograft (CDX) and patient-derived xenograft (PDX) models

For CDX models, 1 × 106 ESCC cells expressing the control shRNA (shCtrl) or TAS1-targeting sh#1 or sh#2 were injected subcutaneously into the dorsal flanks of 4-week-old female BALB/c nu/nu mice (five mice per group). Tumor growth was monitored every 3 days after transplantation using calipers. Mice bearing xenografts were euthanized at the endpoint, and tumor weights were measured. PDX models were constructed as described previously, and fresh ESCC samples obtained from patients were immediately subcutaneously inoculated into both flanks of nude mice. When the first generation of established PDXs (P1) reached ~ 500 mm3, the tumors were cut into pieces of ~ 10 mm3 and transplanted into other mice (P2). Ultimately, mice bearing P3 grafts were used to assess the therapeutic effects of TAS1 in vivo using antisense oligonucleotides (ASOs). When the P3 grafts reached ~500 mm3 at approximately twenty-one days after transplantation, we began to inject 5 nmol of scrambled or in vivo-optimized TAS1 ASOs (RiboBio) per intratumoral injection every 3 days for 4 continuous doses. The target sequence is provided in Supplementary Table 1. Tumor sizes were measured every 3 days just before the next injection. The mice were euthanized at the endpoint, the tumors were dissected, and tumor weights were measured. All CDX and PDX tissues underwent subsequent pathological analyses.

In vivo metastasis models

For the lung metastasis model, 1 × 106 ESCC cells expressing luciferase and transfected with shCtrl or TAS1-targeting sh#1 or sh#2 were intravenously injected into 4-week-old female BALB/c nu/nu mice (six mice per group) through the tail vein. In vivo bioluminescence imaging was performed every four weeks after inoculation. The mice were euthanized 8

weeks after the injection. The lungs were dissected and fixed with 4% paraformaldehyde. Subsequently, consecutive tissue sections were obtained and stained with hematoxylin-eosin (H&E) to observe the metastatic nodules in the lungs under a microscope. The number of lung nodules was measured.

For the inguinal sentinel lymph node metastasis model, 1 × 106 ESCC cells transfected with shCtrl or TAS1-targeting sh#1 or sh#2 were injected into the left footpads of 4-week-old female BALB/c nu/nu mice (six mice per group). Eight weeks after the injection, the mice were euthanized, the corresponding inguinal areas were dissected, and the lymph nodes were collected and fixed with 4% paraformaldehyde. Consecutive slices were made and stained with H&E to observe metastatic micro nodules under a microscope. The metastatic positive lymph node was determined when at least one spot of metastatic micro nodules was observed. The number of metastasis-positive lymph nodes was measured.

Nuclear run-on (NRO) assay

The NRO assay was performed as described. Nuclei from 4×10^6 ESCC cells were freshly isolated with NP-40 lysis buffer and incubated ice before use. Nuclear pellets were resuspended in 40 μl of nuclei storage buffer and used for the transcription assay. Then, the suspended nuclei were incubated with 60 μl of transcription reaction buffer cocktail and 0.1 μmol of BrUTP (Absin, abs42010946) at 30 °C for 30 min. Then, RNAs were isolated with TRIzol Reagent (Invitrogen, USA). The nascent bromouridylated RNA transcripts were immunoprecipitated with 2 μg of an anti-BrdU antibody (Abcam, ab6326) and Protein A/G magnetic beads (MedChemExpress, Shanghai, China) and subjected to quantitative real-time PCR (qPCR) analysis to detect nascent TMPO mRNA expression.

Supplementary Tables

Supplementary Table 1. Sequences of TMPO-AS1 in vivo optimized ASO

ASO name	Target sequence
TMPO-AS1(2OMe+5Chol)	GCAATGGTATTAAGCTCAA

Supplementary Table 2. Primer list

Gene name	Forward	Reverse
TMPO-AS1	CCAGACCCGGACACAAAAGA	CTGCGTTTCTACCTCCTCTCG
ACTB	CTACCTCATGAAGATCCTCACCGA	TTCTCCTTAATGTCACGCACGATT
U6	GCTTCGGCAGCACATATACTAA	TTGCGTGTCATCCTTGCG
18SrRNA	CAGCCACCCGAGATTGAGCA	TAGTAGCGACGGGCGGTGTG
TMPO	CCCCTCGGTCCTGACAAAAG	CGCTCTTCGTCACTGGAGAA
TMPO Promoter1	ACATTTGCCTATGTGTCCAG	GAATCTGAGCTCCAAACTATGTT
TMPO Promoter2	AAAGCCAGAGACGGTTTCAA	GGAAGCCAGA CCTCTACAAT
TMPO Promoter3	GCGCACAAAAGCAGTACGA	CTGAGCGAGA GGAGGTAGA
TMPO Promoter4	TTCGCAGATCCCCGAGATG	TGCAGGTAGAGCTGGACGTACA
METAP2	AAAGGACAAGAATGCGAATACCC	CAGGCTTGATCCAGCTCATTAC
RPL13A	GCCATCGTGGCTAAACAGGTA	GTTGGTGTTCATCCGCTTGC
RB1	CTCTCGTCAGGCTTGAGTTTG	GACATCTCATCTAGGTCAACTGC
PTEN	TGGATTCGACTTAGACTTGACCT	GGTGGGTTATGGTCTTCAAAAGG
RPSA	GTGGCACCAATCTTGACTTCC	GCAGGGTTTTCAATGGCAACAA
DENR	ACAGTGCCAAGTTAGATGCCG	TCCTTGACCCTCACTAATTCCA
CD44	CTGCCGCTTTGCAGGTGTA	CATTGTGGGCAAGGTGCTATT

TNFSF10	TGCGTGCTGATCGTGATCTTC	GCTCGTTGGTAAAGTACACGTA
GNRH1	CAAAAACTCCTAGCTGGCCTT	CAGTTGACCAACCTCTTTGACT
HTATIP2	CGGAGGGATTTGTTCGTGTTG	AGCTCCTTTAGAGGATAGCAAGT
HPRT1	CCTGGCGTCGTGATTAGTGAT	AGACGTTCAGTCCTGTCCATAA
IL18	TCTTCATTGACCAAGGAAATCGG	TCCGGGGTGCATTATCTCTAC
SMAD4	ACGAACGAGTTGTATCACCTGG	TGCACGATTACTTGGTGGATG
NME1	AAGGAGATCGGCTTGTGGTTT	CTGAGCACAGCTCGTGTAATC
MDM2	GAATCATCGGACTCAGGTACATC	TCTGTCTCACTAATTGCTCTCCT
NME2	CCACCTCTTATTCATAGACCCA	AGATTCAAAGCCAGGCACCAT
SET	AGCAAGAAGCGATTGAACACA	TGGTTGGCGGAGTTTGTTATATT
KRAS	ACAGAGAGTGGAGGATGCTTT	TTTCACACAGCCAGGAGTCTT
CCL7	CAAGACCAAACTGGACAAGGAGAT	AGAACCACTCTGAGAAAGGACAGG
SMAD2	CGTCCATCTTGCCATTCACG	CTCAAGCTCATCTAATCGTCCTG
IL1B	ATGATGGCTTATTACAGTGGCAA	GTCGGAGATTCGTAGCTGGA
B2M	GAGGCTATCCAGCGTACTCCA	CGGCAGGCATACTCATCTTTT
ETV4	GATGAAAGCCGGATACTTGGAC	TTCGCGCAAGCTCCCATTT
CXCR4	ACTACACCGAGGAAATGGGCT	CCCACAATGCCAGTTAAGAAGA
CTSK	ACACCCACTGGGAGCTATG	GACAGGGGTACTTTGAGTCCA
MTSS1	CAGTCCCAGCTTCGGACAAC	TGAGAGCAGATCCAATCTCCC
PNN	GTCGCCGTGAGAACTTTGC	GGTCCTCCTCCACTATCTGAGA
CTSL1	CTTTTGCCTGGGAATTGCCTC	CATCGCCTTCCACTTGGTC

VEGFA	AGGGCAGAATCATCACGAAGT	AGGGTCTCGATTGGATGGCA
TIMP4	CCACTCGGCACTTGTGATTC	CATCCTTGACTTTCTCAAACCCT
MET	AGCAATGGGGAGTGTAAAGAGG	CCCAGTCTTGTACTCAGCAAC
MMP7	GAAAGAAATAGAAACTTCAGGCAGA	GAGTGGAGGAACAGTGCTTATC
TRPM1	CAGACAGTAAGTTTTCCATCCC	GAGTACAGTTCAATCACGGACC
MMP10	CAGTAGACAAAGAAGGTAAGGG	AGAGGATAGGCAGAGCAGA
MYCL1	GATGGATGGAGATGTGGAAAT	CGACTCGGAGAATGAAGAAAT
MMP3	GGAGACTTTTACCCTTTTGATGG	TGGTCCCTGTTGTATCCTTTGT
CDKN2A	CCCCGATTGAAAGAACCAGAGAG	TACGGTAGTGGGGGAAGGCATA
RORB	GCTTGATTTCAGTGCTTATTGTGTC	TGGGTCTTCTCTTTCTACCTTTTCT
HPSE	TCTTCCTTGGTAGCAGTCCGT	TTTCATCAATGGGTCGCAGTT
CXCR2	CCCTGCCTGTCTTACTTTTCCGA	ATCCGCCAGTTTGCTGTATTGTTG
APC	CTTCCTCTCCTCATCCAGCTTTTAC	ACGCCTGCCTCTCTTGTCATC
SSTR2	GGACCACCACAAAGTCAAACA	GCTTCCCTTCTACATATTCAACG
HGF	CTCTGGTTCCCCTTCAATAGCAT	TTCCCTTGTAGCTGCGTCCT
FXYD5	GTTTCATCAGCAGGCCAGGTT	ACATTCAGGTCCCGACACGAG
CDH11	CAGATAAAGCAATCTCATGTCTTCC	GTAGCACCAACACCCTCACCA
KISS1	CCTGCCGAACTACAACTGGAAC	TCCCTTAGCCCTACGTCCC
MYC	CGTCCTCGGATTCTCTGCTC	CGATTTCTTCCTCATCTTCTTGTTC
MMP13	ATACTACCATCCTACAAATCTCG	CATCTAAGGTGTTATCGTCAAGT
MGAT5	GGGAACCCAAGTCCAACAAAC	TGAAATCAAAAGGCAGAACCAG
SYK	CTTCACTTCCTTTCATCCCTC	CTAGTTACCCAACATTACGCC

KISS1R	CCGAGACCTGCTGGATGTAGT	ACGTGACCTTCCTCCTGTGCT
CDH1	CAAATCCAACAAAGACAAAGAAGGC	ACACAGCGTGAGAGAAGAGAGT
IGF1	TGTCCTCCTCGCATCTCTTCTACC	CCCCTGTCTCCACACACGAACT
NR4A3	CAGTGGGACAGTATCTGGAATAA	GTCTCAGTGTTGGAATGGTAAA
CDH6	TGGACAACAAATGTACCGACA	AGCTCAAGCTATAAACAGAAGGAC
CXCL12	TCACATCTAACCTCATCTTCTTCAC	ACTCTTCACATAGCACATTGTTCTC
CST7	TCAGTGACAACGGAGAACAGG	GGAGGTGGAAATTGGCAGAAC
TP53	AGCTTTGAGGTGCGTGTTTGTG	TCTCCATCCAGTGGTTTCTTCTTTG
TIMP2	GCTTTCATTCGTCTCCCGTCTTT	CGGCTCTTCTTAACCTGTTTTGTTT
MTA1	TTGTCTGTGAGTGGGTTGTGC	TGTTAAAAGAAGGCGAGGAGG
ITGB3	TTACCTCCTAATTCCACACCCTCAC	CTGGCTCTACAATAGCACTCTCTCC
ITGA7	CCTTGAACTGCTGTCGGTCTT	ACTTGATGCTCCGAGATGCCT
TSHR	CTGGAATCACACTCCTTCTACA	TGGAATAAACTTTGGTCAGGTC
FN1	AAGCCCATAGCTGAGAAGTGTTTTG	GGATGTCCTTGTGTCCTGATCGT
NME4	TGATGTGGACGCTGAAGTCAC	AGGTCTGGGAAGGGTACAATG
GAPDH	GGACCTGACCTGCCGTCTAG	GTAGCCCAGGATGCCCTTGA
CTNNA1	CGTCGCCTCTACCAAATACC	CTTCTGAGATGCCCGTTTA
TIMP3	CTATCGGTATCACCTGGGTTGTA	ATGCAGGCGTAGTGTTTGGA
MMP9	CCACCCTTGTGCTCTTCCCTG	TCTGCCACCCGAGTGTAACCA
FGFR4	ACGAGACTCCAGTGCTGATGG	TCGAATAGGCACAGTTACCCC
MMP11	GGTCTTGGTAGGTGCCTGCATC	CCTCCCCATTTGACTGTGAACTTT
PLAUR	TGGCCGGGCTGTCACCTATT	TTGGACGCCCTTCTTCACCTT

FAT1	TTCTCACCAGTGCCTTTTGTT	TTGAATCCATCCACCCTCCTA
MCAM	TGAGGACTGGCAGTGGAAGTG	CGGCAAGTGAACAAGACCAAG
TGFB1	GAAACCCACAACGAAATCTATGAC	ACGTGCTGCTCCACTTTTAACT
MMP2	AACTACAACTTCTTCCCTCGCAA	CAAAGGCATCATCCACTGTCTCT
FLT4	GATGGTGGTCACATAGAAGTAGAT	TGGAGGGAAAGAATAAGACTGT
CD82	TTCAGTCAGGATGGGCAAGAG	CCATTCCGAAGACTACAGCAA
EPHB2	AACGTGTTTGAGTCAAGCCAGAA	ACGCACCGAAAACTTCATCTCC
TCF20	CGGGTAATGGTATCGGAAGGA	GGTTTGTGGCAGGCTCTATGG
COL4A2	CAACAGAGGACTTGGTTTCTACGGA	TGTACTGATCTGGGTGGAAGGTGA
BRMS1	GCAGTTTGTCATCCCACCATT	GGAGCCTCAAGATTCGCATTC
SRC	ATCACTTCCTTGCCCCCATTTC	CATCCTCAGACCCCTTGTTTCCT
NF2	TGTATCGGGAACCATGATCTATTTA	CTCCATCTGCTTTCTAGCCTTCT
CHD4	AAGTCTTCTTGGTAACTGTGGC	GATCTGACCCCTATTGTGGTAG
HRAS	GTGGAATCTCGGCAGGCTCA	CGCACCAACGTGTAGAAGGCAT
EWSR1	CTGGTAGGAGGGTAGGATGGA	TGGAAACAAGCCCACTGAGAC
CTBP1	AAAGCTGAAGGGTTCCGACTC	CTCAACGAGCACAACCACCAC
ANAPC2	CCTGCGTGAGTTCCACAAGT	GCGGTAGAAGAACCTTTGCAC
CCND1	GCTGCGAAGTGGAAACCATC	CCTCCTTCTGCACACATTTGAA
CCNE1	GCCAGCCTTGGGACAATAATG	CTTGCACGTTGAGTTTGGGT
CDC34	CATCGACTACCCATACTCTCCA	GAGAATGGTCCTGACGTTCTG
CDK4	ATGGCTACCTCTCGATATGAGC	CATTGGGGACTCTCACACTCT
CDK6	CCAGATGGCTCTAACCTCAGT	AACTTCCACGAAAAAGAGGCTT

CDKN1B	AACGTGCGAGTGTCTAACGG	CCCTCTAGGGGTTTGTGATTCT
CDKN3	TCCGGGGCAATACAGACCAT	GCAGCTAATTTGTCCCGAAACTC
CUL1	GATCTGGGACGACCTCAGAG	CCCCTTTTTCGACTTAGAAGGAG
CUL3	GATGCACTGCCTTGACAAATCA	CCTTGCTCCCTCAAATAGGAACT
SKP2	ATGCCCCAATCTTGTCCATCT	CACCGACTGAGTGATAGGTGT

Supplementary Table 3. Sequences of TMPO-AS1 probes for ChIRP

ChIRP Probe	Target sequence
TMPO-AS1 probe#1	AGTACGACCTGTCCCTTATC-/3bio/
TMPO-AS1 probe#2	TTAGGATTCTTGCGGGTGGT-/3bio/
TMPO-AS1 probe#3	CAATAGCCCAACCTCTTAGC-/3bio/
TMPO-AS1 probe#4	GGCAGGAAGGAGAGTAGAAA-/3bio/
TMPO-AS1 probe#5	GTGCCCGATTGTAGAGGTCT-/3bio/
TMPO-AS1 probe#6	CATGGGTCACCTACAAGCAT-/3bio/
TMPO-AS1 probe#7	CCTACATCCAAGGTCTCCTT-/3bio/
TMPO-AS1 probe#8	CCAGTGTTGAGTGCTCCTGA-/3bio/
TMPO-AS1 probe#9	TGGAGCATGGTTTAGTCCAA-/3bio/
Scramble	GCTCCCGTACCGTATTCTAA-/3bio/

Supplementary Table 4. P values of Figure 1b.

			Ctrl	TAS1	HOXC5	HOTAIR	SNHG 11	AGPG	LINC 00941	FAM 225A	LINC 00668
P Value	TE-11	Cell migration	<0.0001	<0.0001	<0.0001	<0.0001	<0.0001	<0.0001	<0.0001	<0.0001	<0.0001
		Cell viability	<0.0001	<0.0001	<0.0001	<0.0001	<0.0001	<0.0001	<0.0001	<0.0001	<0.0001
	KYSE 150	Cell migration	<0.0001	<0.0001	<0.0001	<0.0001	<0.0001	<0.0001	<0.0001	<0.0001	0.0113
		Cell viability	<0.0001	<0.0001	0.0007	<0.0001	<0.0001	<0.0001	<0.0001	<0.0001	0.0057

Supplementary Table 5. Correlation between TMPO-AS1 expression and clinicopathological features in 108 ESCC patients

Variable	low TMPO-AS1 n (%)	high TMPO-AS1 n (%)	*P* value
Total	54(50.0)	54 (50.0)	
Age, years			0.846
>58	30 (55.6%)	31 (57.4%)	
≤58	24 (44.4%)	23 (42.6%)	
Gender			0.835
Female	16 (29.6%)	17 (31.5%)	
Male	38 (70.4%)	37 (68.5%)	
Differentiation status			>0.999
Well/Moderate	45 (83.3%)	45 (83.3%)	
Poor and others	9 (16.7%)	9 (16.7%)	
Tumor depth			0.144
m/sm/mp	20 (37.0%)	13 (24.1%)	
ss/se/si	34 (63.0%)	41 (75.9%)	
Lymph node invasion			0.695
Absent	33 (61.1%)	31 (57.4%)	
Present	21 (38.9%)	23 (42.6%)	
Vascular invasion			0.433
Absent	34 (63.0%)	30 (55.6%)	
Present	20 (37.0%)	24 (44.4%)	
Distant metastasis			0.375
Absent	49 (90.7%)	46 (85.2%)	
Present	5 (9.3%)	8 (14.8%)	
Clinical stage			0.699
I, II	29 (53.7%)	31 (57.4%)	

III, IV	25 (46.3%)	23 (42.6%)

The P value was determined by a Chi-square test. All the statistical tests were two-sided. Abbreviations: m: tumor invasion of mucosa; sm: submocosa; mp: muscularis propria; ss: subserosa; se: serosa penetration; si: invasion to adjacent structures.

Supplementary Table 6: Effect of factors on overall survival in ESCC patients in the univariate and multivariate cox regression model

Factors	univariate		multivariate	
	HR (95% CI)	P value	HR (95% CI)	*P* value
Age	2.004 (1.052-3.816)	0.034	1.960 (1.023-3.753)	0.042
Gender	1.190 (0.856-1.656)	0.301	-	-
Differentiation	1.013 (0.744-1.379)	0.935	-	-
Clinical Stage	3.112 (1.714-5.652)	<0.001	2.792 (1.514-5.148)	0.001
Tumor depth	1.752 (0.945-3.248)	0.075	-	-
Lymph node invasion	2.688 (1.946-3.717)	<0.001	-	-
Vascular invasion	1.272 (0.928-1.742)	0.135	-	-
Distant metastasis	2.006 (1.477-2.882)	<0.001	-	-
TMPO-AS1	2.701 (1.474-4.949)	0.001	2.167 (1.163-4.038)	0.015

The P value was determined by the univariate and multivariate cox regression analysis. All the statistical tests were two-sided.

Supplementary Table 7. Target sequences of ASOs and shRNAs used in this study

**siRNA name**	Target sequence
TMPO-AS1 ASO#1	ACGCAGTTTAAAAGGCGCTG
TMPO-AS1 ASO#2	CTTAGACGCCGATAAGGGAC
TMPO-AS1 shRNA#1	ACGCAGTTTAAAAGGCGCTG
TMPO-AS1 shRNA#2	CTTAGACGCCGATAAGGGAC
TMPO si#1	CCAGGAAGCTATATGAGAA
TMPO si#2	GTGAGTTGGTCGCCAACAA
Negative Control	UUCUCCGAACGUGUCACGUTT

Supplementary Table 8. Gene lists of qPCR array

METAP2	RPL13A	RB1	PTEN	RPSA	DENR	CD44	TNFSF10	GNRH1	HTATIP2	HPRT1	IL18
SMAD4	NME1	MDM2	NME2	SET	KRAS	CCL7	SMAD2	IL1B	B2M	ETV4	CXCR4
CTSK	MTSS1	PNN	CTSL1	VEGFA	TIMP4	MET	MMP7	TRPM1	MMP10	MYCL1	MMP3
CDKN2A	RORB	HPSE	CXCR2	APC	SSTR2	HGF	FXYD5	CDH11	KISS1	MYC	MMP13
MGAT5	SYK	KISS1R	CDH1	IGF1	NR4A3	CDH6	CXCL12	CST7	TP53	TIMP2	MTA1
ITGB3	ITGA7	TSHR	FN1	NME4	GAPDH	CTNNA1	TIMP3	MMP9	FGFR4	MMP11	PLAUR
FAT1	MCAM	TGFB1	MMP2	FLT4	CD82	EPHB2	TCF20	COL4A2	BRMS1	SRC	NF2
CHD4	ACTB	HRAS	EWSR1	CTBP1	ANAPC2	CCND1	CCNE1	CDC34	CDK4	CDK6	CDKN1B
CDKN3	CUL1	CUL2	CUL3	SKP2							

Supplementary Table 9. Correlation between TMPO-AS1/TMPO expression and clinicopathological features in 108 ESCC patients

Variable	TMPO-AS1/ TMPO low n (%)	Intermediate n (%)	TMPO-AS1/ TMPO high n (%)	*P* value
Total	43(39.8%)	22(20.4%)	43(39.8%)	
Age, years				0.222
>58	23(53.5%)	16(72.7%)	22(51.2)	
≤58	20(46.5%)	6(27.3%)	21(48.8%)	
Gender				0.781
Female	12(27.9%)	8(26.4%)	13(30.2%)	
Male	31(72.1%)	14(63.6%)	30(69.8%)	
Differentiation status				0.170
Well/Moderate	39 (90.7%)	16(72.7%)	35(81.4%)	
Poor and others	4(9.3%)	6(27.3%)	7(16.3%)	
Tumor depth				0.781
m/sm/mp	13(30.2%)	8(26.4%)	12(27.9%)	
ss/se/si	30(69.8%)	14(63.6%)	31(72.1%)	
Lymph node invasion				0.511
Absent	23(53.5%)	15(68.2%)	26(60.5%)	
Present	20(46.5%)	7(31.8%)	17(39.5%)	
Vascular invasion				0.599
Absent	28(65.1%)	12(54.5%)	24(55.8%)	
Present	15(34.9%)	10(45.5%)	19(44.2%)	
Distant metastasis				0.967
Absent	38(88.4%)	19(86.4%)	38(88.4%)	
Present	5(11.6%)	3(13.6%)	5(11.6%)	

Clinical stage				0.132
I, II	20(46.5%)	16(72.7%)	24(55.8%)	
III, IV	23(53.5%)	6(27.3%)	19(44.2%)	

The P value was determined by a Chi-square test. All the statistical tests were two-sided.

Abbreviations: m: tumor invasion of mucosa; sm: submocosa; mp: muscularis propria; ss: subserosa; se: serosa penetration; si: invasion to adjacent structures.

Supplementary Table 10. Correlation between TMPO expression and clinicopathological features in 108 ESCC patients

Variable	low TMPO n (%)	high TMPO n (%)	*P* value
Total	54(50.0%)	54 (50.0%)	
Age, years			0.332
>58	33 (61.1%)	28 (51.9%)	
≤58	21 (38.9%)	26 (48.2%)	
Gender			0.531
Female	15 (27.8%)	18 (33.3%)	
Male	39 (72.2%)	36 (66.7%)	
Differentiation status			0.302
Well/Moderate	43 (79.6%)	47 (87.0%)	
Poor and others	11 (20.4%)	7 (13.0%)	
Tumor depth			0.296
m/sm/mp	19 (35.2%)	14(25.9%)	
ss/se/si	35 (64.8%)	40 (74.1%)	
Lymph node invasion			0.117
Absent	28 (51.9%)	36 (66.7%)	
Present	26 (48.2%)	18 (33.3%)	
Vascular invasion			0.240
Absent	35 (64.8%)	29 (53.7%)	
Present	19 (35.2%)	25 (46.3%)	
Distant metastasis			0.139
Absent	50(92.6%)	45 (83.3%)	
Present	4 (7.4%)	9 (16.7%)	
Clinical stage			0.121

I, II	26 (48.2%)	34 (63.0%)
III, IV	28 (51.9%)	20 (37.0%)

The P value was determined by a Chi-square test. All the statistical tests were two-sided.

Abbreviations: m: tumor invasion of mucosa; sm: submocosa; mp: muscularis propria; ss: subserosa; se: serosa penetration; si: invasion to adjacent structures.
